# Exploring the anti-aging potential of natural products and plant extracts in budding yeast *Saccharomyces cerevisiae*: A review

**DOI:** 10.12688/f1000research.141669.1

**Published:** 2023-10-04

**Authors:** Phaniendra Alugoju, Chella Perumal Palanisamy, Naga Venkata Anusha Anthikapalli, Selvaraj Jayaraman, Anchalee Prasanskulab, Siriporn Chuchawankul, Madhu Dyavaiah, Tewin Tencomnao

**Affiliations:** 1 Natural Products for Neuroprotection and Anti-Ageing Research Unit, Chulalongkorn University, Bangkok, 10330, Thailand; 2 Department of Clinical Chemistry, Faculty of Allied Health Sciences, Chulalongkorn University, Bangkok, 10330, Thailand; 3 Department of Chemical Technology, Faculty of Science, Chulalongkorn University, Bangkok, 10330, Thailand; 4 Department of Chemistry, A.N.R College, Gudivada, Andhra Pradesh, 521301, India; 5 Centre of Molecular Medicine and Diagnostics (COMManD), Department of Biochemistry, Saveetha Dental College & Hospital, Saveetha Institute of Medical & Technical Sciences, Saveetha University, Chennai, Tamilnadu, 600077, India; 6 College of Public Health Sciences, Chulalongkorn University, Bangkok, 10330, Thailand; 7 Department of Transfusion Medicine and Clinical Microbiology, Faculty of Allied Health Sciences, Chulalongkorn University, Bangkok, 10330, Thailand; 8 Department of Biochemistry and Molecular Biology, Pondicherry University (A Central University), Puducherry, 605 014, India

**Keywords:** Saccharomyces cerevisiae, Replicative lifespan (RLS), Chronological lifespan (CLS), nutrient signalling pathways, target of rapamycin (TOR), Protein kinase A (PKA), Adenylate cyclase (AC)

## Abstract

Aging is an inevitable multifactorial process associated with a decline in physiological functioning accompanied by a predisposition to a plethora of chronic ailments. Emerging anti-aging research studies using different model organisms have enabled scientists to uncover underlying molecular mechanisms of aging. Notably, the budding yeast *Saccharomyces cerevisiae* has been, and continues to be an indispensable model organism in the field of biomedical research for discovering the molecular causes of aging as well as the anti-aging potential of natural/synthetic compounds and plant extracts. Besides its ease of handling, genetic manipulation, and relatively inexpensive to grow, the budding yeast has preserved nutritional signaling pathways (such as the target of rapamycin (TOR)-Sch9 and the Ras-AC-PKA (cAMP-dependent protein kinase pathways) and two distinct aging paradigms such as chronological life span (CLS) and replicative life span (RLS). In the present review, we have explored the anti-aging properties of several natural products and phytoextracts and their underlying molecular mechanism of action on the CLS and RLS of yeast *S. cerevisiae.*

## Introduction

Aging is an inevitable natural phenomenon characterized by gradual decline in the bodily functions with an increased susceptibility to various internal and external environmental cues and subsequent development of a plethora of chronic diseases, ultimately ending up with death. Aging is considered a major risk factor for the development of several disease conditions including diabetes, cancer, cardiovascular diseases, and neurodegenerative diseases. ^1^^–^^3^ Several hallmarks of aging have been proposed including mitochondrial dysfunction, cell senescence, genome instability, telomere abrasion, epigenetic alterations, malfunction of autophagy, aberrant nutrient-sensing signalling, stem cell dysfunction/exhaustion, and loss of intercellular communication. ^4^^,^^5^ Previous studies have demonstrated the longevity promoting activity of several chemical compounds (known as geroprotectors) to ameliorate hallmarks of aging and to promote healthy lifespan of a variety of model organisms. However, only a very few compounds have been investigated for their potential geroprotective activity in the older people. ^6^ Therefore, the main objective of the biogerontology research is to explore the molecular basis of aging and age-related diseases and to dis-cover new interventions to counteract the detrimental effects of aging and related pathological conditions. ^7^^,^^8^

**Figure 1.**
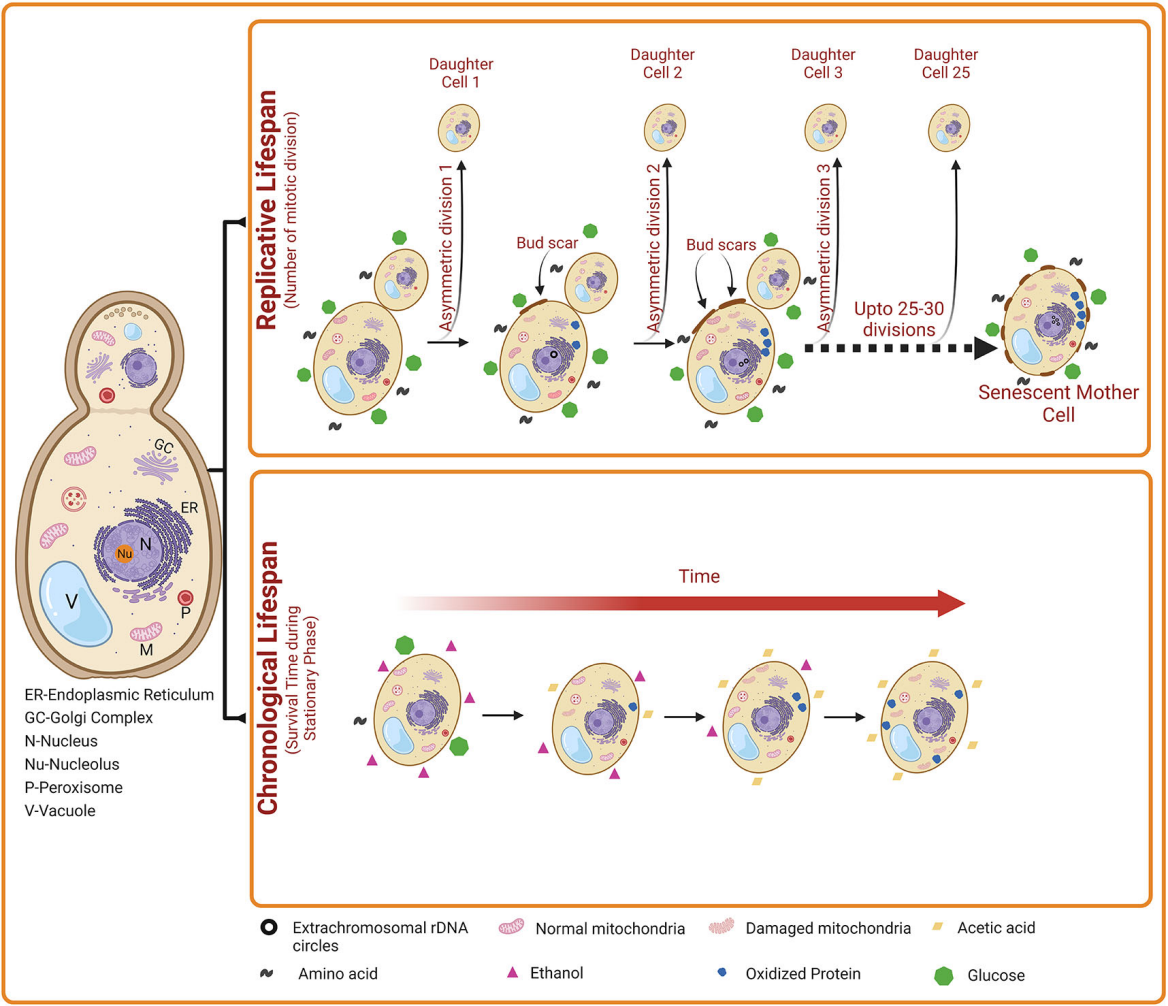
The two aging patterns of *Saccharomyces cerevisiae.* Chronological life span (CLS) indicates the number of days a yeast cell survives in the stationary phase. A gradual decrease in the level of carbon source (e.g., glucose) occurs during stationary phase. Subsequently, ethanol accumulates slowly in the external medium, which is then converted into acetic acid, resulting in the reduction in the pH of the external medium. Elevated acetic acid induces apoptosis of yeast cells. Besides, oxidized proteins and damaged mitochondria also accumulates. Collectively, these events lead to increased cell death with time during yeast Chronological aging. Replicative lifespan (RLS) indicates the number of daughter cells produced by a mother cell. Over time, the accumulation of nuclear extrachromosomal ribosomal DNA circles, oxidized proteins, and damaged mitochondria also occur in the replicatively ageing yeast cells. However, this damage is accumulates only in the mother cells but not in the daughter cells.

**Figure 2.**
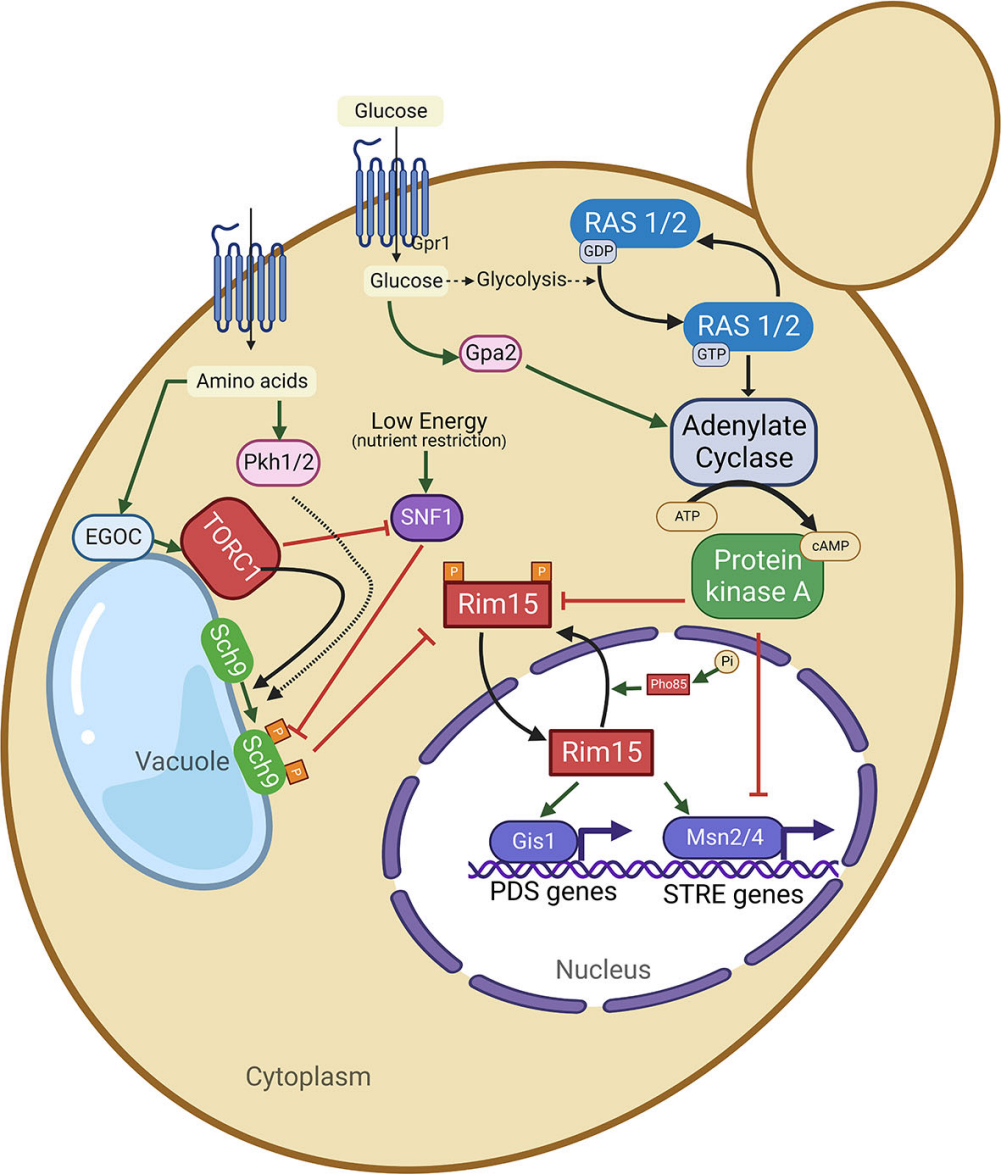
The major nutrient sensing signaling pathways in yeast *Saccharomyces cerevisiae.* Glucose activates adenylate cyclase via stimulation of RAS on one hand and via G protein-coupled receptors (GPCR) system of Gpr1 and Gpa2 on the other hand. The activated adenylated cyclase causes up-surge in the levels of cellular cAMP. Thus, GPR1 and GPA2 are essential for a glucose-dependent rise in the levels of cellular cAMP. Increased intracellular cAMP levels in turn lead to activation of protein kinas A (PKA). On the other hand, cytoplasmic amino acid levels are sensed by EGOC on the vacuolar membrane and this EGOC activates TORC1 which in turn activates Sch9 via phosphorylation. TORC1 can inhibit SNF1, which acts as an energy sensor. Both SNF1 and PKH1/2 can influence the activity of Sch9. Both the nutrient signaling pathways RAS-AC-PKA and TORC1-Sch9 converge at Rim15 protein kinase. The activated protein kinases PKA and Sch9 (of RAS-AC-PKA and TORC1-SCH9 signaling pathways) phosphorylate and inhibit Rim15 protein kinase activity, resulting in the inhibition of its transport to the nucleus. When both PKA and Sch9 are inactive, Rim15 (unphosphorylated form) gets activated and imported to the nucleus, where it induces the activation of transcription factors such as Msn2/4 and Gis1 leading the respective transcription of stress-responsive (STRE) genes and postdiauxic shift (PDS) genes. On the other hand, within the nucleus, Rim15 is also inactivated via phosphorylation by a Pho85 cy-clin-dependent kinase (in association with its cyclin partner Pho80). Phosphorylated Rim15 is exported to from the nucleus to the cytoplasm via the Msn5 receptor protein.

Globally, there is an increase in the aging population and concomitant increase in the chronic diseases affecting the quality of life of elderly. ^9^ This leads to rising treatment costs and economic burden. Therefore, understanding of the aging phenomenon and its underlying molecular mechanisms are crucial to ameliorate age-related disease progression, to delay aging or promote active health lifespan of elderly people. ^10^ Aging research using different model organisms from simple eukaryotic yeast to mammalian models led to the discovery of several genes and molecular pathways involved in aging of humans. ^11^ Owing to their short lifespan, ease of genetic manipulation, and relatively inexpensive to maintain in the laboratory, several model organisms such as *Caenorhabditis elegans* (nematode), *Drosophila melanogaster* (fruit fly), *Mus musculus* (mouse), *Rattus norvegicus* (rat), and the budding yeast *Saccharomyces cerevisiae* have been used not only for the understanding of aging and age-related diseases, but also for the discovery of several anti-aging compounds. ^11^ Nonetheless, these model organisms have served as potential tool for the discovery and evaluation of a wide spectrum of the pharmacological properties including anti-aging potential of several phytochemicals. ^12^^,^^13^ In this review, we have summarized the anti-aging effects of natural products and plants on the budding yeast *Saccharomyces cerevisiae.*

## The budding yeast *Saccharomyces cerevisiae* – A simple eukaryotic model to study aging

The budding yeast *S. cerevisiae*, also known as baker’s or brewer’s yeast, is a single-celled eukaryotic organism composed of several membrane-bound organelles similar to animal cells including a nucleus, endoplasmic reticulum, Golgi complex, vacuole, cytoskeleton, mitochondria, and other different organelles. ^14^ Yeast cells are round to ovoid in shape with a size of ∼5 μm in diameter (unbudded cell), between bacteria and human cells in size. Yeast cells divide once every 90 min under optimal laboratory conditions, through a process of budding in which smaller daughter cells detach from their mother cell. The budding yeast was the first eukaryotic organism whose genome was completely sequenced and released in 1996. The haploid yeast cell contains 16 chromosomes comprising about ∼12,068 kb of genomic DNA. The genome of yeast is thought to be evolved from the whole-genome duplication of its ancestral set of 8 distinct chromosomes. ^15^ The *S. cerevisiae* yeast genome is composed of many genes that can be grouped into protein-coding genes (5885) and non-coding genes. The budding yeast nuclear genome is composed of more than 6,600 open reading frames (ORFs). The yeast genome is also comprised of 786 dubious ORFs which probably do not encode any proteins. Interestingly, the yeast genome has a very low number of introns, approximately 4% of all genes, as a result of which, the yeast genome is composed of a high number of protein-coding genes (one gene every 2 kbp). The non-coding genes in yeast are transcribed into transfer RNA (tRNA), ribosomal RNA (rRNA), small nuclear RNA (snRNA), and small nucleolar RNA (SnoRNA). ^15^ The best advantages of yeast as a model are that it is easy to handle, its short generation time, and ease of genetic manipulation. Most interestingly, yeast share several homologous and orthologous genes with mammals including humans, as a result of which yeast can be used even to study human diseases. The budding yeast has long been used as a model organism for the identification of the molecular basis of several cellular processes including cell cycle, autophagy, protein folding, oxidative stress, and aging.

## Replicative lifespan (RLS) and chronological lifespan (CLS)

Yeast exhibit two distinct patterns of aging such as chronological lifespan (CLS) and replicative lifespan (RLS). The RLS measures the mitotic potential of a single yeast cell ( *i.e.*, how many bud cells are generated from a single mother cell). Thus, yeast RLS is similar to the mitotic cell division of mammalian cells. For the first time in 1959, Robert Mortimer and John Johnston discovered the aging phenomenon in budding yeast. They reported that yeast cells can divide asymmetrically through mitosis (budding) for a limited number of divisions (~25) and then stop dividing. ^16^ As the mother cell divides by mitosis, it accumulates molecular damage; however, the daughter cells retain replicative capacity but generally do not inherit such damage from the mother cell. However, some studies on yeast replicative aging have suggested the asymmetric inheritance of at least three different types of damage, including nuclear extrachromosomal ribosomal DNA (rDNA) circles, oxidatively damaged or misfolded cytoplasmic proteins, and dysfunctional mitochondria. ^17^ Phenotypically, old yeast mother cells are larger in size than the daughters and carry a number of bud scars indicating yeast’s RLS. In old mother cells, the actin cytoskeleton is found to be distorted and therefore, mother cells need longer times to complete the cell cycle compared to daughter cells which show a normal dotted and chain-type cytoskeleton. In addition, mother cells also accumulate high levels of mitochondrial-derived reactive oxygen species (ROS) and apoptotic features such as externalization of phosphatidyl serine, nuclear DNA fragmentation and chromatin marginalization.

On the other hand, the CLS measures the yeast cell viability in the non-dividing phase ( *i.e.*, postmitotic phase). Thus, CLS is similar to the postmitotic aging of mammalian cells. The CLS is defined as the time duration a yeast cell can survive in a nondividing state, with survival determined by the ability to reenter the cell cycle and resume vegetative growth upon exposure to appropriate growth-promoting cues. ^17^^,^^18^ Chronological aging is also characterized by the accumulation of protein carbonyl content and dysfunctional mitochondria. In worms, flies and mice, the key regulators of CLS also control RLS and aging. In addition, chronologically aged cells also show a reduction in subsequent RLS, suggesting that similar forms of age-associated damage may contribute to both mitotic (RLS) and postmitotic (CLS) aging in yeast cells. ^19^ During CLS, the size of yeast cells is normal and they enter a stationary phase due to starvation. The stationary phase cells are characterized by altered cellular metabolism towards the synthesis of reserved carbohydrates such as glycogen and trehalose. During CLS, the starving yeast cells utilize glycogen whereas trehalose is used for membrane stabilization and other non-metabolic functions. In addition, stationary phase cells show a hard and thick cell wall. Stationary phase yeast cells exhibit heterogeneity and can be categorized into quiescent (G0) and non-quiescent cells. Quiescent cells described as unbudded daughter cells form only during the final cell division in the diauxic phase of the growth curve, and have a high density compared to normal yeast cells. In addition, quiescent cells can reenter synchronously the mitotic cell cycle. In contrast, non-quiescent cells are less dense and composed of heterogenous, asynchronous, and replicatively older cells that lose their division capacity. Non-quiescent cells accumulate elevated ROS levels compared to quiescent cells and exhibit apoptotic as well as necrotic features. After a longer time, the quiescent cells also start to show landmarks of apoptosis, and finally of necrosis. ^20^ Though the phenotypes of replicatively older yeast cells (in RLS) and stationary phase yeast cells (in CLS) are different, features such as augmented levels of ROS and intracellular oxidative stress, apoptosis and necrosis are found to be common.

## Conserved nutrient sensing aging regulatory pathways in budding yeast

Though budding yeast exhibits different aging paradigms, two major nutrient sensing pro-aging signaling pathways, such as the TOR-Sch9 and the RAS-AC-PKA pathways, promote aging and early death in both RLS and CLS, thus playing a similar role in both aging paradigms, whereas SIR2 does not. ^21^ Thus, the inhibition or inactivation of the RAS-AC-PKA pathway results in the extension of both CLS and RLS in yeast. ^21^ RAS proteins act as molecular switches depending on their GTP-bound or unbound state. The yeast *RAS2* gene is homologous to human RAS proto-oncogene. In yeast, the aging regulator pathways such as protein kinase A (PKA) and mitogen-activated protein kinase (MAPK) pathways are dependent on the activation of RAS2. Activated PKA inhibits the activity of two important transcription factors, Msn2/4, involved in yeast stress resistance. It has been reported that the deletion of *RAS2* gene (that encodes Ras2, a G-protein) results in increased stress resistance, thereby increasing the yeast lifespan. However, deletion of *MSN2/MSN4* genes reverts the phenotype observed with *RAS2* gene deletion. In yeast, transcription factors such as Msn2/4 and Gis1 bind respectively to the STRE (STress Responsive Element) and the PDS (post-diauxic shift) sequences, thereby activating stress resistance genes including *SOD1 SOD2*, *CTT1*, *HSPs*, and *DDR2.* However, activated protein Kinase A (PKA) inhibits Rim15, a PAS-kinase that integrates signals from different nutrient sensing signaling pathways, including TORC1, Sch9, PKA, and Pho80-Pho85, and subsequently affects different transcription factors, Msn2/4 and Gis1. ^22^ The inhibition of Rim15 positively regulates the transcription factors such as Msn2/4 and Gis1. It is hypothesized that Rim15 also plays a role in the regulation of genes involved in the glucose repression and cell cycle arrest. ^22^

Sch9 is a serine/threonine protein kinase homologous to mammalian Akt (a serine/threonine kinase) and S6K (S6 kinase). On the other hand, deletion of *SCH9* can extend both RLS and CLS of yeast. In addition, the combined deletion of *SCH9* along with *RAS2* can further extend the yeast CLS compared to the deletion of SCH9 alone. Both CLS and RLS can be extended by inhibiting or deleting the serine/threonine-protein kinase TOR1, via inactivating the downstream Sch9 protein kinase. Tor and Sch9 lead to the inactivation of the serine/threonine kinase Rim15 and the stress resistance transcription factor Gis1, both of which are required for maximum chronological life span extension. ^21^ Similar to Msn2 and Msn4, Gis1 induces the expression of mitochondrial superoxide dismutase enzyme (MnSOD), which is required for the effect of SCH9 deletion on the CLS. Aging yeast is characterized by elevated levels of ROS. In contrast, reduced levels of ROS have been observed in the long-lived mutants deficient in Ras-AC-PKA or TOR-Sch9 signaling.

During CLS, glucose levels decrease gradually and accumulation of non-fermentable carbon sources such as ethanol and acetic acid takes place. Both ethanol and acetic acid promote the CLS. Loss of either TOR1or SCH9 slows down the aging process partly by lowering respiration and stimulating the exhaustion of ethanol and acetic acid and the elevating the levels of glycerol in the nutrient medium. Since *S. cerevisiae* cannot ferment either acetic acid or ethanol, their depletion by mutations in *TOR* or *SCH9* can prolong the CLS by creating similar conditions usually caused by dietary restriction. It is important to note that the yeast cells deficient in TOR-Sch9 or Ras-AC-PKA signaling pathways exhibit prolonged CLS during incubation in water or in a medium which is either deficient or composed of low amounts of acetic acid and ethanol. ^22^^,^^23^

## Anti-aging studies conducted using the budding yeast *S. cerevisiae* as a model

### Acetyl L-carnitine

Acetyl-L-carnitine (ALC) is an endogenous molecule synthesized in the body from L-carnitine. ALC plays a key role in the energy metabolism and it has been reported to exhibit a plethora of pharmacological properties, including neuroprotective activities. It has also been used as a dietary supplement due to its potent therapeutic effects. Researchers have shown the anti-apoptotic and anti-aging ability of ALC in yeast model. ALC was found to inhibit mitochondrial fission and subsequently improved mitochondrial functioning. Furthermore, it was found that the mitoprotective effects of ALC were mediated through the yeast metacaspase (Yca1) and thus to its anti-apoptotic activity. ALC was also shown to extend the CLS of yeast cells. ^24^

### Amarogentin

Disasa *et al.* isolated a secoiridoid glycoside amarogentin from a Chinese traditional medicinal plant, *Gentiana rigescens* Franch. Amarogentin was reported to enhance both the enzymatic activities and expression levels of superoxide dismutase (SOD), catalase (CAT), and glutathione peroxidase (GPx), thereby enhancing the viability of yeast cells exposed to oxidative stress. Furthermore, treatment with amarogentin resulted in an increased RLS of yeast wild type; however, this compound was not shown to enhance the lifespan of yeast mutants *sod1Δ*, *sod2Δ*, *uthΔ*, and *skn7Δ*, suggesting that the anti-aging effects of amarogentin are mainly through the regulation of antioxidative stress as well as by the regulation of *UTH1*, *SKN7*, *SOD1*, and yeast *SOD2* gene expression. ^25^

### Astaxanthin

Astaxanthin is a carotenoid compound with superior antioxidant ability than many other natural antioxidant molecules. Sudarshan *et al.* conducted a study to test the antioxidant, anti-apoptotic and anti-aging properties of astaxanthin using yeast model. Astaxanthin treatment prevented oxidative stress-induced elevation in MDA and ROS levels and reduction in superoxide dismutase and glutathione levels, thereby increasing the percentage viability of antioxidant gene-deleted yeast mutants by 20-40%. In addition, astaxanthin also prevented the apoptosis of aged cells and subsequently increased the viability of yeast cells during the CLS. Astaxanthin’s antioxidant and anti-apoptotic effects are the possible reason for its anti-aging activity. ^26^ It was also suggested that anti-apoptotic effects of astaxanthin are also due to its ability to prevent nuclear fragmentation and chromatin condensation in yeast cells. ^27^ Sudarshan *et al*. also found that astaxanthin enhanced the oxidative stress resistance and increased the viability of yeast DNA damage repair gene-deleted mutant cells. Astaxanthin was shown to prevent the accumulation of endogenous DNA damage marker (8-hydroxy-2-deoxyguanosine) levels in yeast cells. The anti-aging effects of astaxanthin were also suggested to be due to its ability to prevent the accumulation of mutation during chronological aging of DNA damage repair gene-deleted mutant yeast cells. ^27^

### Artesunate

Artesunate is a semi-synthetic derivative of the antimalarial drug artemisinin. Previous studies have shown that artesunate exerts anti-aging effects similar to caloric restriction, indicating the CR mimetic effects of artesunate. From the whole-transcriptome profile analysis studies, it was revealed that artesunate mimics CR-triggered nitric oxide to induce antioxidant defense systems, thereby preventing ROS accumulation and mitigated oxidative stress, subsequently extending yeast lifespan. ^28^

### Betulinic acid

Betulinic acid (BA) is a pentacyclic triterpenoid present in some plant species. A recent study reported that betulinic acid extends the chronological life span of yeast cells by mitigating oxidative stress-induced apoptosis. The anti-aging effects of betulinic acid were found to be mediated by the activation of genes associated with heat shock stress response and autophagy. ^29^

### Almond extracts

Almond ( *Prunus dulcis* (Mill.) D.A. Webb) is a major nut crop worldwide. Multiple health benefits associated with the consumption of almonds are due to the phenolic-rich almond skin. Previous studies have found that pre-treatment with almond skin extract and chlorogenic acid significantly enhanced the lifespan of yeast. Both almond extract and chlorogenic acid improved the mitochondrial function during chronological aging of yeast cells by reducing the accumulation of free radicals (including ROS and RNS) and by preserving mitochondrial membrane potential. Furthermore, treatment with almond extract and chlorogenic acid increased oxidative stress response by inducing the expression levels of *SIR2* and *SOD1* genes and by decreasing the endogenous levels of lipid peroxides, protein carbonyls and 8-hydroxy-2-deoxyguanosine in yeast cells. Altogether, it can be suggested that the longevity-extending effects of almond extract are ascribed to its ability to ameliorate oxidative stress in yeast cells. ^30^

### Citrus flavonoids

Flavonoids have been reported to exert a plethora of pharmacological activities including antioxidant, anti-inflammatory, anti-cancer, anti-neurodegenerative, anti-aging, among others. Researchers have investigated the anti-aging effects of citrus flavonoids including naringin, hesperedin, hesperitin, and neohesperidin on the chronological aging of yeast. Among the tested flavonoids, neohesperidin significantly prevented accumulation of ROS and extended the chronological life span of yeast in a concentration-dependent manner. ^31^ In another study, treatment with hesperidin significantly increased the expression levels of SOD and sirtuin2 (SIR2) and prevented ROS accumulation in yeast. In yeast, *UTH1* gene encodes Uth1p, which is activated by oxidative stress, senescence, and TOR-dependent autophagy; all these events subsequently lead to cell death in yeast. Pretreatment with hesperidin, but not its aglycon hesperetin, significantly inhibited *UTH1* gene expression, thereby extending yeast lifespan. ^32^

### Curcumin

Curcumin is a biologically active yellow-colored carotenoid compound with potent anti-oxidant and anti-aging properties. Previous studies investigated the replicative and chronological life span-extending effects of curcumin using yeast. It was demonstrated that curcumin significantly increased oxidative stress and enhanced both replicative and chronological life span of yeast mutants that lacked antioxidant genes ( *SOD1* and *SOD2*) and DNA damage repair gene *RAD52.* Overall, it can be suggested that curcumin exerts anti-aging hormetic effects in yeast. ^33^

### Cucurbitacins

Cucurbitacins are a class of tetracyclic triterpenoids that are abundant in plants belonging to the family Cucurbitaceae. Cucurbitacins have been reported to exhibit strong anticancer activity, and are divided into 12 classes from A to T with over 200 derivatives. ^34^ Cucurbitacin B is the most abundant and active member of the cucurbitacins. ^35^ Researchers have discovered that cucurbitacin B can extend both replicative and chronological life spans in yeast. Treatment with cucurbitacin B increased the lifespan of yeast by promoting the expression of *ATG32.* Cucurbitacin B could not increase the lifespan of yeast mutants devoid of autophagy related genes ( *ATG2* and *ATG32*), pointing to the fact that the anti-aging effects of cucurbitacin B are mainly through the induction of autophagy in yeast. It was also demonstrated that cucurbitacin B can ameliorate oxidative stress levels mainly through the induction of superoxidase dismutase activity (via increase expression of *SOD* and *SOD2*) and prevention of accumulation of oxidative stress markers including ROS and malondialdehyde (MDA). Furthermore, researchers also showed that the anti-aging effects of cucurbitacin B are also mediated through the regulation of expression of other aging-related genes such as *UTH1* as well as *SKN7.* Cucurbitacin B’s anti-aging effects are attributed to its ability to ameliorate oxidative stress and regulation of autophagy, and age-related genes in yeast. ^36^ Additionally, cucurbitane glycoside isolated from the methanol extracts of *Momordica charantia* L. fruits has been reported to significantly extend the RLS of K6001 budding yeast mainly through suppressing the ROS levels and oxidative stress burden. Besides, treatment with cucurbitane glycoside resulted in a decrease in the expression levels of *UTH1* and *SKN7* and increase in the expression levels of *SOD1* and *SOD2.* However, cucurbitane glycoside was not able to extend the RLS of the yeast mutants of *uth1Δ*, *skn7Δ*, *sod1Δ*, and *sod2Δ*, suggesting that cucurbitane glycoside exerts antiaging effects via antioxidative stress and regulation of yeast *UTH1*, *SKN7*, *SOD1*, and *SOD2* gene expression. ^37^

### 4-N-furfurylcytosine

Previous studies have demonstrated that purine and pyrimidine derivates can exhibit promising health promoting effects. ^38^^,^^39^ Pawelczak *et al.* investigated the antiaging effects of 4-N-Furfurylcytosine (FC), a cytosine derivative using model *S. cerevisiae.* They found that treatment with FC enhanced the percentage viability of yeast cells during CLS in a concentration-dependent manner. In addition, treatment with FC boosted the mitochondrial activity and abridged intracellular levels of ROS. It was also demonstrated that FC could limit TORC1 (target of rapamycin complex 1) signaling in yeast. This points to the fact that FC’s anti-aging activity is through the inhibition of TOR signaling pathway. ^40^

### Gentirigeoside B and gentiopicroside

Gentirigeoside B is a triterpenoid glycoside isolated from the Chinese traditional medicinal plant *Gentiana rigescens* Franch. Xiang, L., *et al.* reported that gentirigeoside B significantly extended both the replicative and chronological lifespan of yeast. It was also demonstrated that treatment with gentirigeoside B augmented the activity of antioxidant enzymes (including superoxide dismutase, catalase, and glutathione peroxidase) and abridged the oxidative stress markers (ROS and MDA), thereby preventing oxidative stress-induced reduction in the viability of yeast cells. Gentirigeoside-B treated cells also showed downregulation of Sch9 and activation of Rim15 and Msn2 proteins. This indicates that the anti-aging potential of gentirigeoside B is due to its ability to inhibit the TORC1/Sch9/Rim15/Msn signaling. However, gentirigeoside B could not enhance the life span of yeast mutant lacking antioxidant genes ( *sod1∆*, *sod2∆*, *cat1∆*, and *gpx∆*) and age-related genes ( *skn7∆* and *uth1∆*). Therefore, Xiang, L., *et al.* suggested that though treatment with gentirigeoside B might have longevity-promoting effects in humans, the effect may not be effective in those with mutations in endogenous antioxidant enzyme genes. ^41^ In another study, gentiopicroside, a secoiridoid glycoside that was also isolated from *G. rigescens* Franch was reported to extend both RLS and CLS of yeast. Gentiopicroside was shown to induce ATG32 gene expression, but could not prolong the RLS and CLS of yeast mutants deficient in ATG32 gene. It was also reported that gentiopicroside enhanced the yeast survival rate under oxidative stress condition by augmenting the activities of enzymatic antioxidants and diminishing the accumulation of ROS and lipid peroxidation. However, gentiopicroside could not affect the RLSs of *sod1Δ*, *sod2Δ*, *uth1Δ*, and *skn7Δ.* Overall, it can be suggested that gentiopicroside’s anti-aging effects are attributed to its ability to ameliorate autophagy and antioxidative stress response. ^42^

### Galactan exopolysaccharide

A recent study using yeast models investigated the antioxidant and anti-aging properties of galactan exopolysaccharide isolated from *Weissella confusa.* Galactan exopolysaccharide was shown to prevent the oxidative stress induced elevation of ROS levels and enhanced the viability of yeast cells exposed to hydrogen peroxide as an oxidant used in this study. In addition, galactan exopolysaccharide treatment significantly increased the viability of both wild type and antioxidant gene-deleted mutant ( *sod2∆*). The study suggested that the antioxidant potential of the galactan exopolysaccharide could be the possible reason for its anti-aging activity. ^43^

### Ganodermasides A and B

Medicinal mushrooms have been reported to exhibit anti-aging activity. In a study, ^38^ researchers isolated two ergosterol derivatives, namely ganodermasides A and B, from the spores of the medicinal mushroom *Ganoderma lucidum.* They showed that both ganodermasides A and B could extend the replicative life span of *S. cerevisiae* through the modulation of the expression of UTH. In yeast, different transcription factors such as Skn7, Yap1, and Mot3 play a crucial role in oxidative stress resistance. It was suggested that upon phosphorylation, these transcription factors get activated, thereby regulating the expression of *UTH1* gene by binding to its upstream promoter region. It was reported that polyphenols’ anti-aging activity occurs through the activation of Skn7 and subsequent increased expression of the *UTH1* gene. ^44^

### Ginsengosides

Ginsenosides are major pharmacological compounds that are unique to the plant species *Panax ginseng* C. A. Meyer (ginseng). Ginsenosides have been demonstrated to increase the life span of different model organism. In a recent study, ^39^ researchers treated yeast cells with ginsenoside Rg1 and found an increased antioxidant stress response and concomitant reduction in ROS levels and apoptosis, thereby enhancing visibility during aging in yeast model. It was also demonstrated that ginsenoside Rg1 treatment resulted in enhanced mitochondrial bioenergetics and glycolytic enzymes, thereby improving metabolic homeostasis and delayed aging in *S. cerevisiae.* ^45^

### Quercetin

In a previous study, ^46^ we demonstrated the protective effects of quercetin on yeast *S. cerevisiae pep4Δ* mutant that lack the *PEP4* gene (that encodes vacuolar endopeptidase proteinase A, which is a homolog of the human cathepsin D). Yeast *pep4Δ* cells are found to be highly sensitive to oxidative and apoptotic stressors. However, in our study, we demonstrated that quercetin treatment reduced the ROS levels and apoptotic markers, thereby enhancing the percentage viability of yeast *pep4Δ* cells during chronological aging. ^46^ Mutation in ATM gene is linked to increased oxidative damage, premature aging, and apoptosis. In another study, we also investigated the protective effects of quercetin on the sensitivity of *S. cerevisiae tel1Δ* cells lacking Tel1p, which is a homolog of the human *ATM* (Ataxia Telangiectasia Mutated) gene mutation. Our study results showed that quercetin treatment prevented ROS accumulation and thereby enhanced the stress resistance of *tel1Δ* cells exposed to a variety of oxidizing agents. Furthermore, treatment with quercetin prevented apoptotic death of yeast *tel1Δ* cells and increased cell viability during chronological aging. ^47^

### Coffee

Czachor, J., *et al.* demonstrated the lifespan extending effects of coffee infusions in *S. cerevisiae.* Coffee, especially the *Coffea robusta* type, was found to exhibit superior antioxidant effects than the *Coffea arabica* type, thereby protecting cells from ROS-induced DNA damage and additionally ameliorating the metabolic activity in yeast cells. overall, it can be suggested that coffee exhibits health benefits mainly though the amelioration of ROS accumulation and metabolic activity. ^48^

### Glutamic acid and methionine

A study ^43^ reported that the composition of amino acids in aging media can affect the chronological life span of yeast cells. The presence of non-essential amino acids methionine and glutamic acid have been reported to greatly influence the survival rate of yeast cells during aging. Precisely, low levels of methionine and high levels of glutamic acid in the yeast nutrient media led to enhanced lifespan in yeast model. In contrast, increasing levels of methionine and reducing levels of glutamic acid caused a decrease in yeast life span. Therefore, it can be concluded that amino acid composition is a critical factor for controlling the yeast aging. ^49^

### Rapamycin

Eukaryotic cells growth and proliferation is regulated by an evolutionarily conserved kinase, the target of rapamycin (TOR). It has been shown that the inactivation of TOR signaling pathway leads to lifespan extension in different eukaryotic model organisms. The eukaryotic nucleolus is rich in ribosomal DNA (rDNA) that is composed of multiple tandem repeats of rRNA genes (100-200 copies rDNA repeats) and the components for ribosome assembly. Inside the nucleolus, the transcription of rDNA produces precursor rRNA that in turn will be processed further and become associated with ribosomal proteins to form preribosomal subunits. Sir2 is a histone deacetylase enzyme involved in silencing the transcription at the rDNA locus to enhance yeast life span. ^50^^,^^51^ Nearly 50% of the rDNA repeats are maintained in a silent state partly by the Sir2 protein. ^52^ Using *S. cerevisiae* yeast as model, it was found that rapamycin treatment cause inhibition of the TORC1 complex, resulting in the increased association of Sir2 with ribosomal DNA (rDNA) in the nucleolus. This association of SIR2 with rDNA reduces homologous recombination between rDNA repeats that causes formation of toxic extrachromosomal rDNA circles. Thus, rapamycin treatment-induced TORC1 inhibition signals the stabilization of rDNA locus by promoting the association of Sir2 with rDNA, thereby extending the RLS in *S. cerevisiae.* ^53^

### Spermidine

Spermidine has been reported to inhibit histone acetyltransferases and subsequent deacetylation of histone H3, thereby preventing oxidative stress and necrosis in yeast aging. On the contrary, reduction of endogenous polyamines caused hyperacetylation, accumulation of ROS, necrotic cell death and diminished life span. It is important to note that treatment with spermidine causes alteration in acetylation status of the chromatin which results in substantial increase in the autophagy in different model organisms, including yeast. This suggests that spermidine’s anti-aging effects are mediated through its ability to stimulate the autophagy pathway. ^54^

### Copper and iron supplementation

Previous studies ^49^^,^^50^ were conducted to demonstrate the effects of supplementation of copper and iron on the yeast RLS. It was found that addition of copper to the growth media increased the RLS via the activation of multicopper oxidase enzyme (Fet3p) that works in combination with another enzyme called iron permease (Ftr1p) that allows the high intake of iron into yeast cells. Furthermore, it is important to note that high levels of iron-mediated anti-aging effects are dependent on the multicopper oxidase enzyme. However, the life span-extending effects of both copper and iron occurred in the growth medium supplemented with only glycerol as a carbon source but not glucose. It was also demonstrated that supplementation of either copper or iron prevented the accumulation of ROS levels, thereby enhancing the replicative life span of yeast mutants lacking antioxidant enzymes. ^55^^,^^56^ The iron-mediated anti-aging effects occur mainly through the increased expression of genes related to the mitochondrial metabolism especially tricarboxylic acid (TCA) cycle and electron transport chain, subsequently elevated levels of adenosine triphosphate (ATP), which is essential for the increased survival cells during aging. Interestingly, supplementation of iron could also increase the life span of yeast *Snf1Δ* mutant which lacks SNF1/AMPK protein kinase. Therefore, it can be suggested that the iron’s life span-extending effects occur via amelioration of mitochondrial energy metabolism in yeast. ^57^

### Inokosterone

A recent study ^58^ isolated a compound inokosterone from *G. rigescens* Franch and demonstrated that inokosterone can increase both the chronological and replicative life spans. The inokosterone was shown to enhance the survival rate of yeast cells by ameliorating the levels of antioxidant enzymes (e.g., SOD) and preventing accumulation of oxidative stress markers (e.g., ROS and MDA levels). Further, inkosterone could increase the autophagy (especially mitophagy) in yeast cells. Likewise, the same study also demonstrated that treatment with inkosterone decreased the oxidative stress and enhanced autophagy in mammalian cell lines. Therefore, it can be suggested that inkosterone’s anti-aging effects are mediated through the activation of antioxidant stress response and mitophagy. ^58^

### Lithocholic acid

Previous studies have reported that lithocholic acid, a bile acid, can increase yeast cell survival during chronological aging. ^59^^–^^62^ Interestingly, lithocholioc acid was also shown to significantly enhance the survival rate of yeast especially under caloric restriction conditions. Lithocholic acid treatment was shown to modulate several cellular pathways, including carbohydrate and lipid metabolism, mitochondrial structure and function, liponecrotic and apoptotic cell death of yeast during chronological aging. ^59^ It is important to note that lithocholic acid’s anti-aging effects under caloric restriction are found to be prominent only when this compound is added to the growth medium either at logarithmic/diauxic and early stationary stages of yeast aging. Addition of lithocholic acid either at logarithmic/diauxic and early stationary stages induced the activation of several longevity related cellular processes which ultimately triggering the enhanced yeast cell survival during aging. ^60^ Beach *et al.* also demonstrated that lithocholic acid exerts its anti-aging effects through the modulation of the expression of different transcription factors including Aft1p, Hog1p, Msn2/4p, Rtg1p- Rtg3p, Sfp1p, Skn7p, and Yap1p. Each of these transcription factors in turn alter the levels of several intra and extra mitochondrial proteome, and subsequent maintenance of mitochondrial function. Altogether, Beach *et al.* suggested that lithocholic acid’ anti-aging effects are mainly through the modulation of aging-related transcriptional landscape. ^61^ Also, it was discovered that lithocholic bile acid accumulates in mitochondria, and alters the mitochondrial membrane lipidome which is crucial for the restoration of mitochondrial proteome, subsequently an improved mitochondrial function and increased chronological aging by lithocholic acid. ^62^

### Lysophosphatidic acid

In another study, Sun Y *et al.*, isolated lysophosphatidic acid (LA) from the seeds of *Arabidopsis thaliana* and showed that LA increased oxidative stress resistance, thereby promoting the extension of RLS in yeast. However, LA was not able to extend the replicative lifespan of mutants *uth1Δ*, *skn7Δ*, *sod1Δ*, and *sod2Δ.* This study suggests that LA’s anti-aging effects are possibly through the amelioration of antioxidant status of yeast cells and the genes of *UTH1*, *SKN7*, and *SOD* may also be involved in the action. ^63^

### Magnolol

In our previous study, ^58^ we reported the anti-aging effects of magnolol, a natural polyphenol. Magnolol increased the stress resistance of yeast cells exposed to hydrogen peroxide, an oxidizing agent. In addition, magnolol increased the viability of short-lived yeast mutant *sod1∆*, which lacks the antioxidant enzyme superoxide dismutase. Our study suggested that the anti-aging effects of magnolol occur mainly through the modulation of oxidative stress during yeast chronological aging. ^64^

### Morusin and mulberrin

Mulberry leaves are rich in flavonoids such as morusin and mulberrin, and these have been demonstrated to increase survival rate during yeast chronological aging. It was also found that morusin and mulberrin exert their anti-aging effects via targeting the SCH9, a major target of TORC1 in budding yeast *S. cerevisiae.* ^65^ In eukaryotes, cell growth is regulated by two different, highly conserved multiprotein complexes such as TORC1 and TORC2. These complexes are comprised of a large catalytic subunit, the serine/threonine kinase target of rapamycin (TOR). Budding yeast Sch9 is an AGC (Protein Kinase A (PKA), G (PKG), and C (PKC)) family kinase and is the major substrate of the TORC1. Yeast Sch9 functions analogously to S6K1, the mammalian TORC1 substrate. Yeast TORC1 phosphorylates about six amino acid residues of Sch9, indicating that the TORC1-dependent phosphorylation of Sch9 is essential for its activity. ^66^ However, rapamycin treatment, carbon and nitrogen starvation inhibit phosphorylation by TORC1. Activated Sch9 plays a key role in the regulation of several cellular processes including protein synthesis (via modulating ribosome synthesis and translation initiation), cell cycle, and aging. Deletion of SCH9 causes growth defects such as reduction in cell size and growth rate; however, deletion of SCH9 leads to enhanced temperature tolerance as well chronological and replica life spans in budding yeast. The longevity-extending effects due to SCH9 deletion are explained by the augmented oxidative stress response and reduction in age-associated mutation rate. ^67^

### Phloridzin

Xiang, L. *et al.*, investigated the anti-aging effects of apple polyphenol, phloridzin using yeast RLS model. Phloridzin treatment significantly increased the percentage viability of yeast cells exposed to hydrogen peroxide. It was also demonstrated that phloridzin treatment led to increased expression of SOD1/2 and SIRT1 genes as well as the activity of superoxide dismutase. Altogether, it was suggested that the anti-aging effects of phloridzin are mediated through the regulation of expression of SIRT1 in budding yeast. ^68^

### Polyalthia longifolia

*Polyalthia longifolia* is a polyphenol-rich traditional medicinal plant that has long been used for its rejuvenation capacity. *P. longifolia* has been reported to exhibit potent pharmacological activities including antioxidant and hepatoprotective activities. A previous study ^63^ tested the anti-aging activity of methanolic leaf extract of *P. longifolia* using yeast CLS model and found that the methanolic leaf extract enhanced the viability of yeast cells and increased the CLS of yeast. It was reported that methanolic leaf extract significantly prevented the accumulation of H _2_O _2_-induced increase in ROS levels and augmented the GSH levels. Furthermore, treatment with methanolic leaf extract strikingly enhanced the expression levels of *SOD* and *SIRT1* genes. Overall, it can be suggested that *P. longifolia* exerts its anti-aging effects through the modulation of oxidative stress response and *SIRT1* gene. ^69^ It was also reported that methanolic leaf extract of *P. longifolia* extended the RLS of yeast via ameliorating the apoptotic features of replicatively ageing yeast cells. ^70^

### Myricetin

In another study, using budding yeast as a model, researchers investigated antioxidant and anti-aging effects of myricetin, a polyphenolic flavonoid compound which is composed of a pyrogallol ring in the B ring of its structure. Myricetin’s antioxidant effects are attributed to the presence of a hydroxyl group in its B ring. Treatment with myricetin reduced the augmentation of ROS and protein carbonyl levels, thereby increasing the oxidative stress resistance of yeast cells exposed to hydrogen peroxide. Myricetin was found to inhibit H _2_O _2_-induced glutathione oxidation, but did not increase endogenous antioxidant enzymatic activity in yeast. Additionally, myricetin treatment significantly prevented age associated oxidative stress and enhanced the chronological lifespan of yeast mutant lacking mitochondrial superoxide dis-mutase ( *sod2Δ*). ^71^

### Nicotinamide adenine dinucleotide (NAD+) precursors

Alterations in the biosynthesis and regulation of nicotinamide adenine dinucleotide (NAD+) plays a crucial role in the progression of aging as well as pathophysiology of several age-related chronic diseases. ^72^ NAD + plays a crucial role in several metabolic processes in the cells. Most importantly, NAD+ functions as a co-factor in redox reactions and it is reduced to NADH in many metabolic pathways including glycolysis, the citric acid cycle, and fatty acid metabolism. Adequate intracellular levels of NAD+ are maintained through the combined action of NAD(+) biosynthesis and salvage pathways. Several key cellular enzymes including sirtuin protein deacetylases and poly-ADP-ribose polymerases (PARPs) are NAD+ dependent, and use NAD+ as a substrate in many cellular processes, including aging. Aging is associated with a reduction in NAD+ levels, leading to alterations in several metabolic processes that are NAD+-dependent, which in turn leads to the ageing-associated physiological/functional decline. Therefore, supplementation with diets rich in three important NAD+ precursors 1) nicotinamide, 2) nicotinic acid ( *e.g.*, protein rich foods such as cereals, peanuts, meat, fish) and 3) nicotinamide riboside ( *e.g.*, milk, cabbage, cucumber) ameliorate age-related decline in NAD+ levels and help in the extension of active longevity. ^73^ Nicotinamide riboside is a natural product present in milk. ^13^ NAD+ is also required for the proper functioning of Sir2 and extension of replicative life span in the budding yeast. Alterations in NAD+ levels negatively affect the RLS of yeast. In eukaryotes, nicotinamide riboside kinases (Nrk1 and Nrk2) catalyze the phosphorylation of nicotinamide riboside to nicotinamide mononucleotide, the precursor of NAD+. A previous study reported that the supplementation of nicotinamide riboside increased the levels of NAD+ via activation of both Nrk1-dependent pathway and the Urh1/Pnp1/Meu1 pathway (an Nrk1-independent pathway). Increased NAD+ levels in turn lead to increased Sir2-dependent gene silencing, thereby extending RLS in yeast. ^74^ The Sir2 enzyme deacetylates the lysine residues of histones, thereby silence all heterochromatin-like regions including telomeres, rDNA, and the hidden mating type loci HML/HMR (Hidden MAT Left/ (Hidden MAT Right). ^75^ In the *S. cerevisiae* NAD+ salvage pathway, nicotinamide is recycled from NAD+ by the Sir2 mediated deacetylation reaction. The nicotinamidase (Pnc1) catalyzes the conversion of nicotinamide to nicotinic acid, which is further converted to nicotinamide mononucleotide by the enzyme nicotinamide phosphoribosyltransferase (Npt1). Isonicotinamide (a compound similar in shape and electrical properties to nicotinamide) was shown to elevate the intracellular levels of NAD+ through the yeast NAD+ salvage pathway and concomitant increase in the Sir2 activation resulting in the enhanced normal silencing at the rDNA locus in yeast and extension of yeast RLS. ^76^

### Resveratrol

Mitochondrial dynamics, the balance between mitochondrial fission and fusion, is critical for cell growth and functioning. Replicative senescence in yeast is characterized by the presence of fragmented mitochondria due to mitochondrial fission rather than fusion, indicating altered mitochondrial dynamics. Wang *et al.* reported that treatment with resveratrol led to a significant reduction in the number of senescent yeast cells with fragmented mitochondria. This indicates that resveratrol’s anti-aging effects possibly occur via modulating the expression of genes associated with mitochondrial dynamics during RLS. ^77^

### Rice bran extract

Pigmented rice is the functional food in many countries including India, China, and Japan, and it is the richest source of polyphenols. ^78^ It has been reported that red rice bran extract could prevent the accumulation of ROS levels, maintain the plasma membrane integrity and extend the chronological lifespan of yeast cells. ^79^ The anti-aging mechanism of action of rice bran extract was found to be through the modulation of TOR1 and SIR2-dependent pathway. ^79^

### Sacred lotus stamen extract

The stamen of lotus ( *Nelumbo nucifera*) is rich in flavonoids and has long been widely used in Indian traditional medicine. Tungmunnithum *et al.* reported that treatment with lotus stamen extract significantly increased the antioxidant status (both enzymatic activity and gene expression levels of SOD1 and SIRT2) and extended the chronological lifespan of yeast cells. Interestingly, the longevity extending effects of lotus stamen extract were reported to be superior to the resveratrol treatment. ^80^

### Tanshinones

Wu *et al*. found that the dried roots of *Salvia miltiorrhiza* Bunge have substantial longevity extending effects. They reported that tanshinones ( *e.g.*, cryptotanshinone, tanshinone I, and tanshinone IIa) are pharmacologically active components present in the roots of *S. miltiorrhiza.* Precisely, cryptotanshinone has been reported to extend the CLS of yeast wild type as well as yeast mutant lacking mitochondrial superoxide dismutase ( *sod2Δ*). Their study suggests that the cryptotanshinone-induced anti-aging effects are modulated through the involvement of nutrient-sensing protein kinases such as Tor1, Sch9, and Gcn2. ^81^

### Parishin

Parishin is a phenolic glucoside isolated from *Gastrodia elata*, a Chinese traditional medicinal plant. Parishin treatment significantly increased cell viability in oxidative stress and also extended the replicative life span of K6001 yeast. Parishin treatment significantly increased the expression of levels of SIR2 and SOD activity, while preventing the accumulation of ROS and lipid peroxidation. However, Parishin treatment did not increase the RLS of *sod1Δ*, *sod2Δ*, *uth1Δ*, and *skn7Δ* mutants of K6001 yeast. Treatment with parishin resulted in the reduced expression of several TOR signaling pathway-related genes such as TORC1, ribosomal protein S26A (RPS26A), and ribosomal protein L9A (RPL9A). In addition, parishin treatment significantly diminished gene expression levels of *RPS26A* and *RPL9A* in *uth1Δ* mutant, as well as in *uth1 Δsir2Δ* double mutants. Also, parishin treatment remarkably inhibited the TORC1 gene expression in uth1 mutants. These study results indicate that parishin exerts its anti-aging activities by modulating Sir2/Uth1/TOR signaling pathway. ^82^

### Psoralea corylifolia

*P. corylifolia* is a medicinal plant widely used in the traditional medicine systems in Indian and China. Wang *et al.* showed that the ethanol extract of *P. corylifolia* significantly increased the RLS of yeast. In particular, the n-hexane fraction of the ethanol extract of *P. corylifolia* improved the yeast lifespan by 20%. Interestingly, the n-hexane extract of *P. corylifolia* extended the mean lifespan of the *sir2Δfob1Δ* double mutant strain, indicating that the n-hexane fraction prolongs the RLS in a Sir2-independent pathway. Treatment with *P. corylifolia* did not extend the RLS of the *tor1Δ* mutant, suggesting that Tor1 plays a major role in the extension of RLS by the n-hexane-soluble fraction *P. corylifolia.* Furthermore, it was reported that, Corylin and neobavaisoflavone are the active compounds in *P. corylifolia* that significantly increased the viability and extended the RLS of yeast. In particular, corylin was found to be more effective in enhancing the yeast RLS compared to neobavaisoflavone. It was also shown that corylin treatment promoted RLS of the *sir2Δfob1Δ* mutant strain but failed to prolong the RLS of the *tor1Δ* mutant, suggesting that corylin exerts life span-extending effects in a Tor1-dependent pathway. Fascinatingly, under CR conditions, corylin could not extend the RLS. In addition, corylin treatment did not significantly influence the CLS of yeast. Gtr1 in *S. cerevisiae* encodes a highly conserved GTPase that is necessary for TORC1 activation and amino acid sensing. The G protein complex Gtr1/Gtr2 activates Tor1 in *S. cerevisiae* in response to signals from amino acids. ^83^ Furthermore, it was also suggested from docking studies that corylin prolongs the yeast RLS by blocking Gtr1 activation. ^84^

Previous studies by Lee MB et al ^85^ also demonstrated that green tea extract and berberine could strongly shorten the yeast life span, whereas *Pterocarpus marsupium* extract and other mixtures containing *P. marsupium* significantly extended the yeast life span. ^85^ In another study, two sesquiterpene glucosides isolated from the Shenzhou honey peach fruit were also able to extend the RLS of K6001 yeast. Treatment with sesquiterpene glucosides increased the survival rate of yeast under oxidative stress. Besides, treatment with sesquiterpene glucosides could not affect the RLSs of SOD mutant yeast strains with a K6001 background, indicating that the anti-oxidative stress response performs important roles in anti-aging effects of sesquiterpene glucosides. ^86^

## Conclusions

Aging is a significant risk factor for the emergence of several chronic human diseases. In order to reduce the pathophysiology of diseases related to aging and to increase the active lifetime of people, aging research is primarily concerned with the identification of new anti-aging therapies. Budding yeast *S. cerevisiae *has been used as a valuable model to evaluate the anti-aging properties of phytochemicals as well as synthetic compounds. Several studies demonstrated that plant extracts/phytochemicals can increase longevity via regulation of various pathways including the TORC1/Sch9/Rim15/Msn signaling pathway, RAS-AC-PKA pathway, Sir2-dependent pathway, NAD+-salvage pathway, Uth1/TOR signaling, and antioxidant stress response pathway as well as autophagy. While many of these studies reported enhancing the yeast cell’s oxidative stress resistance, a few studies have also documented the pro-oxidant effects of various antioxidants, suggesting dose-dependent effects on life span. For example, in the K6001 yeast strain of *S. cerevisiae*, treatment with alpha-tocopherol and coenzyme Q10 enhanced oxidative stress and shortened RLS, showing the potential pro-oxidant effects of antioxidants that may be dose-dependent. ^87^ Therefore, to maximize the therapeutic benefits of antioxidants, it is necessary to evaluate their dose-dependent effects as well as potential pro-oxidant actions. The discovery of novel plant-derived chemicals with anti-aging activities opens up new opportunities for developing leading medications for the treatment of age-related disorders.

In conclusion, aging research using budding yeast as a model organism has yielded valuable insights into the anti-aging properties of various compounds, and the identification of specific pathways and mechanisms involved contributes to the development of potential anti-aging therapies for improving health span and longevity in humans. Continued research into plant extracts and natural compounds may lead to the identification of promising candidates for fighting age-related disorders in the future.

## Data Availability

No data are associated with this article.
