# Exploring the anti-aging potential of natural products and plant extracts in budding yeast *Saccharomyces cerevisiae*: A review

**DOI:** 10.12688/f1000research.141669.2

**Published:** 2024-12-17

**Authors:** Phaniendra Alugoju, Chella Perumal Palanisamy, Naga Venkata Anusha Anthikapalli, Selvaraj Jayaraman, Anchalee Prasanskulab, Siriporn Chuchawankul, Madhu Dyavaiah, Tewin Tencomnao

**Affiliations:** 1 Department of Clinical Chemistry, Faculty of Allied Health Sciences, Chulalongkorn University, Bangkok, 10330, Thailand; 2 Natural Products for Neuroprotection and Anti-Ageing Research Unit, Chulalongkorn University, Bangkok, 10330, Thailand; 3 Department of Chemical Technology, Faculty of Science, Chulalongkorn University, Bangkok, 10330, Thailand; 4 Department of Chemistry, A.N.R College, Gudivada, Andhra Pradesh, 521301, India; 5 Centre of Molecular Medicine and Diagnostics (COMManD), Department of Biochemistry, Saveetha Dental College & Hospital, Saveetha Institute of Medical & Technical Sciences, Saveetha University, Chennai, Tamilnadu, 600077, India; 6 College of Public Health Sciences, Chulalongkorn University, Bangkok, 10330, Thailand; 7 Department of Transfusion Medicine and Clinical Microbiology, Faculty of Allied Health Sciences, Chulalongkorn University, Bangkok, 10330, Thailand; 8 Department of Biochemistry and Molecular Biology, Pondicherry University (A Central University), Puducherry, 605 014, India

**Keywords:** Saccharomyces cerevisiae, Replicative lifespan (RLS), Chronological lifespan (CLS), nutrient signalling pathways, target of rapamycin (TOR), Protein kinase A (PKA), Adenylate cyclase (AC)

## Abstract

Historically, plant derived natural products and their crude extracts have been used to treat a wide range of ailments across the world. Biogerontology research aims to explore the molecular basis of aging and discover new anti-aging therapeutic compounds or formulations to combat the detrimental effects of aging and promote a healthy life span. The budding yeast *Saccharomyces cerevisiae* has been, and continues to be, an indispensable model organism in the field of biomedical research for discovering the molecular basis of aging *S. cerevisiae* has preserved nutritional signaling pathways (such as the target of rapamycin (TOR)-Sch9 and the Ras-AC-PKA (cAMP-dependent protein kinase) pathways, and shows two distinct aging paradigms chronological life span (CLS) and replicative life span (RLS). This review explores the anti-aging properties of natural products, predominantly derived from plants, and phytoextracts using *S. cerevisiae* as a model organism.

## Introduction

Aging is an inevitable natural phenomenon characterized by gradual decline in the bodily functions with an increased susceptibility to various internal and external environmental cues and subsequent development of a plethora of chronic diseases, ultimately ending up with death. Aging is considered a major risk factor for the development of several disease conditions including diabetes, cancer, cardiovascular diseases, and neurodegenerative diseases. ^1^^–^^3^ Several hallmarks of aging have been proposed including mitochondrial dysfunction, cell senescence, genome instability, telomere abrasion, epigenetic alterations, malfunction of autophagy, aberrant nutrient-sensing signalling, stem cell dysfunction/exhaustion, and loss of intercellular communication. ^4^^,^^5^ Globally, there is an increase in the aging population and concomitant increase in the chronic diseases affecting the quality of life of elderly. ^6^^,^^7^ Currently existing anti-aging strategies, such as dietary or caloric restriction (CR), exercise, and exogenous bioactive supplements, have shown to be promising in delaying or preventing chronic diseases and promoting active longevity in humans. ^8^^,^^9^ However, life style associated anti-aging strategies like CR, exercise, and improved sleep quality alone may not be sufficient to delay aging and prevent age-related comorbidities, highlighting the need for the development of additional strategies, such as dietary supplements based on natural products from medicinal plants. ^9^

Since ancient times, humans have been exploring natural products, including plants, not only for their food needs but also for the treatment of various ailments. Historically, medicinal plants have been used in various therapeutic formulations in the form crude extracts, decoctions, and gels. Plants have formed the basis of diverse traditional medicinal systems, including Indian Ayurveda, Chinese, Egyptian, and Unani medicine. The oldest records dating back to around 2600 BCE, document the use of about 1000 plant derived substances, such as oils, for the treatment of various ailments like cough, colds, infections, and inflammations in Mesopotamia. These oils were extracted from plant species including *Cedrus, Cupressus sempevirens, Glycyrrhiza glabra*, and others. Around 1500 BCE, it was documented in Papyrus Ebers that more than 700 plants derived drugs were used in Egyptian medicine. ^10^ By 1100 BCE, the Chinese Materia Medica recorded about 1,215 plan-based drugs. ^11^ Likewise, Sushruta and Samhita have documented 857 herbal drugs in Ayurveda. ^12^

According to the World Health Organization (WHO), about 65% of the global population depends on the traditional plant-based medicine for primary healthcare needs. It has been reported that approximately 80% of the pure compounds that are used as drugs were derived from only 94 plant species. ^13^ Moreover, a variety of derivatives of pure compounds are developed as drugs for treating different human diseases. For instance, an alkaloid compound, galegine, isolated from *Galega officinalis* formed the basis for the synthesis of anti-diabetic drugs such as metformin and other biguanide derivatives. ^14^ Likewise, papaverine, isolated from *Papaver somniferum,* formed the basis for the synthesis of drugs like verapamil for treating hypertension, and pain relief drugs morphine and codeine. ^14^^,^^15^

It is no surprise that traditional plant-based medicine has always played a critical role in discovering and developing several drugs to cure human diseases. For example, in the early 1600s, the bark of *Cinchona* species was used to treat fevers in Amazon and Europe. In 1820, the antimalarial alkaloid compound quinine was isolated from the bark of *Cinchona officinalis,* ^15^ forming the basis for the synthesis of other anti-malarial drugs such as chloroquine and mefloquine. Likewise, *Artemisia annua* has been widely used to treat fevers in Traditional Chinese Medicine. In 1972, an antimalarial drug artemisinin was isolated from *A. annua.* ^16^ Other clinically important drugs developed from medicinal plants include, the anti-hypertensive drug reserpine from *Rauwolfia serpentina*, the anti-asthmatic drug ephedrine from *Ephedra sinica,* anticancer drugs such as vinblastine and vincristine from *Catharanthus roseus* and, and paclitaxel from *Taxus baccata.* ^17^^–^^19^ Despite the extensive scientific exploration of terrestrial plants, only 6% of about 300, 000 plant species have been systematically examined, pharmacologically, and only 15% were investigated phytochemically. ^20^

Several natural product-based products have also been approved by the United States Food and Drug Administration (FDA). For example, Veregen™, a topical ointment for genital warts consisting of a mixture of green tea catechins, was approved in 2006. ^21^ Sativex ^®^, a neuropathic pain-relieving formulation composed of cannabis plant derived dronabinol 1 and cannabidol 2, was approved in 2005. ^22^ It was also approved in 2007 for use as an analgesic for cancer patients. ^23^ Qutenza ^®^, a transdermal patch containing capsaicin, an active component of hot chili peppers, was approved in 2009 and has also been used against neuropathic pain. ^24^ This point to the fact that natural products or their derivatives has always been attracted a great attention owing to their ability to serve as templates for the invention and development of therapeutically active formulations or drugs against multiple chronic illness.

Previous studies have demonstrated the longevity promoting activity of several chemical compounds (known as geroprotectors) to ameliorate hallmarks of aging and to promote healthy lifespan of a variety of model organisms. However, only a very few compounds have been investigated for their potential geroprotective activity in the older people. ^25^ The main objective of the biogerontology research is to explore the molecular basis of aging and age-related diseases and to discover new interventions to counteract the detrimental effects of aging and related pathological conditions. ^26^^,^^27^ The budding yeast *Saccharomyces cerevisiae* is one of the widely used model organisms not only for the understanding of aging and age-related diseases, but also as a potential tool for the discovery and evaluation of a wide spectrum of the pharmacological properties including anti-aging potential of several natural products and plant extracts. ^28^^–^^30^ In this review, we have summarized the anti-aging effects of natural products, mostly plant derived and plant extracts in the budding yeast *S. cerevisiae.*

## The budding yeast *Saccharomyces cerevisiae* – A simple eukaryotic model to study aging

The budding yeast *S. cerevisiae*, also known as baker’s or brewer’s yeast, is a single-celled eukaryotic organism composed of several membrane-bound organelles similar to animal cells including a nucleus, endoplasmic reticulum, Golgi complex, vacuole, cytoskeleton, mitochondria, and other different organelles. ^31^ Yeast cells are round to ovoid in shape with a size of ∼5 μm in diameter (unbudded cell), between bacteria and human cells in size. Yeast cells divide once every 90 min under optimal laboratory conditions, through a process of budding in which smaller daughter cells detach from their mother cell. The budding yeast was the first eukaryotic organism whose genome was completely sequenced and released in 1996. The haploid yeast cell contains 16 chromosomes comprising about ∼12,068 kb of genomic DNA. The genome of yeast is thought to be evolved from the whole-genome duplication of its ancestral set of 8 distinct chromosomes. ^32^ The *S. cerevisiae* yeast genome is composed of many genes that can be grouped into protein-coding genes (5885) and non-coding genes. The budding yeast nuclear genome is composed of more than 6,600 open reading frames (ORFs). The yeast genome is also comprised of 786 dubious ORFs which probably do not encode any proteins. Interestingly, the yeast genome has a very low number of introns, approximately 4% of all genes, as a result of which, the yeast genome is composed of a high number of protein-coding genes (one gene every 2 kbp). The non-coding genes in yeast are transcribed into transfer RNA (tRNA), ribosomal RNA (rRNA), small nuclear RNA (snRNA), and small nucleolar RNA (SnoRNA). ^32^

The best advantages of yeast as a model are that it is easy to handle, its short generation time, and ease of genetic manipulation. Most interestingly, yeast share several homologous and orthologous genes with mammals including humans, as a result of which yeast can be used even to study human diseases. The budding yeast has long been used as a model organism for the identification of the molecular basis of several cellular processes including cell cycle, autophagy, protein folding, oxidative stress, and aging. These features make yeast an ideal model organism for the high-throughput screening of identifying genes and chemical compounds associated with aging. However, it is important to note that, its unicellular nature limits its ability to model complex multicellular organisms and tissue-specific aging processes in humans. Additionally, some post-translational modifications that occur in humans do not occur in yeast, and yeast metabolism significantly differs from humans. These features may limit the application of certain findings to human aging research.

## Replicative lifespan (RLS) and chronological lifespan (CLS)

**Figure 1.**
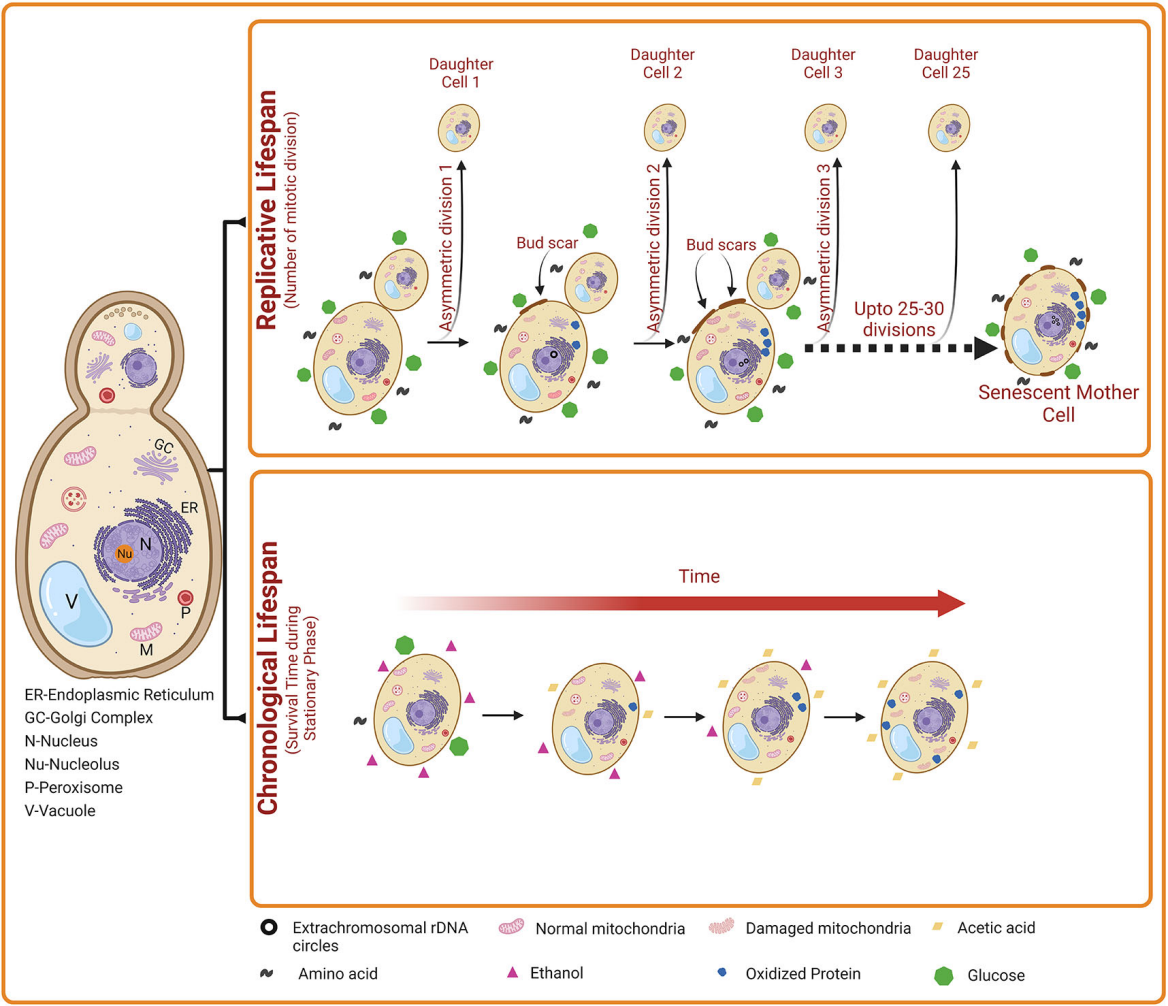
The two aging patterns of *Saccharomyces cerevisiae.* Chronological life span (CLS) indicates the number of days a yeast cell survives in the stationary phase. A gradual decrease in the level of carbon source (e.g., glucose) occurs during stationary phase. Subsequently, ethanol accumulates slowly in the external medium, which is then converted into acetic acid, resulting in the reduction in the pH of the external medium. Elevated acetic acid induces apoptosis of yeast cells. Besides, oxidized proteins and damaged mitochondria also accumulates. Collectively, these events lead to increased cell death with time during yeast Chronological aging. Replicative lifespan (RLS) indicates the number of daughter cells produced by a mother cell. Over time, the accumulation of nuclear extrachromosomal ribosomal DNA circles, oxidized proteins, and damaged mitochondria also occur in the replicatively ageing yeast cells. However, this damage accumulates only in the mother cells but not in the daughter cells.

Yeast exhibit two distinct patterns of aging such as chronological lifespan (CLS) and replicative lifespan (RLS) ( Figure 1). The ***Replicative Lifespan (RLS)*** measures the mitotic potential of a single yeast cell ( *i.e.*, how many bud cells are generated from a single mother cell). Thus, yeast RLS is similar to the mitotic cell division of mammalian cells. For the first time in 1959, Robert Mortimer and John Johnston discovered the aging phenomenon in budding yeast. ^33^ They reported that yeast cells can divide asymmetrically through mitosis (budding) for a limited number of divisions (~25) and then stop dividing ( Figure 1). As the mother cell divides by mitosis, it accumulates molecular damage; however, the daughter cells retain replicative capacity but generally do not inherit such damage from the mother cell. However, some studies on yeast replicative aging have suggested the asymmetric inheritance of at least three different types of damage, including nuclear extrachromosomal ribosomal DNA (rDNA) circles, oxidatively damaged or misfolded cytoplasmic proteins, and dysfunctional mitochondria ( Figure 1). ^34^ Phenotypically, old yeast mother cells are larger in size than the daughters and carry a number of bud scars indicating yeast’s RLS. In old mother cells, the actin cytoskeleton is found to be distorted and therefore, mother cells need longer times to complete the cell cycle compared to daughter cells which show a normal dotted and chain-type cytoskeleton. In addition, mother cells also accumulate high levels of mitochondrial-derived reactive oxygen species (ROS) and apoptotic features such as externalization of phosphatidyl serine, nuclear DNA fragmentation and chromatin marginalization.

Accumulation of ERCs is considered as the one of the best characterized and important hall marks of RLS. These ERCs are generated due to homologous recombination within rDNA which leads to the formation of circular DNA molecules with self-replicating capacity. Therefore, molecules or proteins that target rDNA homologous recombination and ERC formation affect the RLS. In yeast, a NAD+ dependent histone deacetylase which is encoded by the silent information regulator 2 (SIR2) gene, suppresses ERC formation by inhibiting rDNA homologous recombination. In contrast, the fork block protein (Fob1p) encoded by FOB1 gene promote rDNA recombination and ERCs formation. Therefore, the overexpression of SIR2 or the deletion of FOB1 increases RLS, while deletion of SIR2 gene reduces RLS. ^35^ Although, exact underlying pro-aging mechanisms of ERCs are yet to known, it is speculated that the necessary replication machinery including DNA polymerases, helicase, primase as well as the other transcription factors might interact physically with the accumulating ERCs, resulting in the acceleration of RLS. Another plausible mechanism is increased transcription of rDNA to rRNA, subsequently the imbalance between rRNA and ribosomal proteins ultimately leading to the impaired ribosomal synthesis and function. Also, it is suggested that elevated levels of ERCs induce rDNA stability promoting replicative aging. ^36^

In addition to the prominent role of ERCs in modulating RLS, other factors such as the accumulation of oxidatively modified (i.e., carbonylated) proteins and dysfunctional mitochondria contribute to the progression of replicative aging in yeast. The carbonylated proteins distribute asymmetrically in a Sir2 dependent manner between mother and bud cells, with bud cells not inheriting the oxidized proteins. The daughter cells receive higher levels of undamaged, yet active, cytosolic catalase Ctt1p after cytokinesis, which substantially diminishes ROS levels and attenuates oxidative stress. ^37^^,^^38^ The beta subunit of mitochondrial F1F0 ATP synthase (Atp2), encoded by ATP2 gene, is essential for the segregation of mitochondria between mother and daughter cells during budding process. Any mutations in ATP2 gene or its deletion not only affects the proper distribution of mitochondria between mother and daughter cells but also induce mitochondrial dysfunction, ultimately influencing the RLS. ^39^ Beside SIR2, RTG2 is another important gene involved in the longevity in yeast. It encodes the Rtg2 (ReTroGrade 2), which acts as a sensor of mitochondrial dysfunction. Rtg2 plays a key role in the mitochondrial retrograde signaling pathway, which relays signals from mitochondria to nucleus. Activation of this pathway by dysfunctional mitochondria leads to changes in nuclear gene expression to restore cellular homeostasis, resulting in the extension of RLS. Deletion of RTG2 enhances the formation of ERCs thereby negative affects the RLS. In contrast, overexpression of RTG2 reduces ERC formation and extends RLS in yeast. ^40^

The ***Chronological life span (CLS)*** measures the yeast cell viability in the non-dividing phase ( *i.e.*, postmitotic phase). Thus, CLS is similar to the postmitotic aging of mammalian cells. The CLS is defined as the time duration a yeast cell can survive in a nondividing state ( Figure 1), with survival determined by the ability to reenter the cell cycle and resume vegetative growth upon exposure to appropriate growth-promoting cues. ^34^^,^^35^ Chronological aging is also characterized by the accumulation of protein carbonyl content and dysfunctional mitochondria. In worms, flies and mice, the key regulators of CLS also control RLS and aging. In addition, chronologically aged cells also show a reduction in subsequent RLS, suggesting that similar forms of age-associated damage may contribute to both mitotic (RLS) and postmitotic (CLS) aging in yeast cells. ^41^ During CLS, the size of yeast cells is normal and they enter a stationary phase due to starvation. The stationary phase cells are characterized by altered cellular metabolism towards the synthesis of reserved carbohydrates such as glycogen and trehalose. During CLS, the starving yeast cells utilize glycogen whereas trehalose is used for membrane stabilization and other non-metabolic functions. In addition, stationary phase cells show a hard and thick cell wall. Stationary phase yeast cells exhibit heterogeneity and can be categorized into quiescent (G0) and non-quiescent cells. ^42^ Quiescent cells described as unbudded daughter cells form only during the final cell division in the diauxic phase of the growth curve, and have a high density compared to normal yeast cells. In addition, quiescent cells can reenter synchronously the mitotic cell cycle. In contrast, non-quiescent cells are less dense and composed of heterogenous, asynchronous, and replicatively older cells that lose their division capacity. Non-quiescent cells accumulate elevated ROS levels compared to quiescent cells and exhibit apoptotic as well as necrotic features. After a longer time, the quiescent cells also start to show landmarks of apoptosis, and finally of necrosis. ^42^ Though the phenotypes of replicatively older yeast cells (in RLS) and stationary phase yeast cells (in CLS) are different, features such as augmented levels of ROS and intracellular oxidative stress, apoptosis and necrosis are found to be common.

In a standard CLS experiment, yeast cells are grown in either a synthetic defined medium or a nutrient-rich YPD (yeast extract, peptone, and dextrose) medium with glucose as a carbon source. ^43^ During exponential phase, yeast cells proliferate by fermenting glucose to ethanol, leading to the accumulation of ethanol in the initial growth phase. After glucose is depleted, growth rate decreases as cells temporarily arrest growth to adjust their metabolism from fermentation to the respiration. ^44^ This shift in metabolism known as diauxic shift, involves cells slowly using secondary carbon sources like ethanol, which is converted to acetic acid, resulting in the acidification of the growth medium. After the ethanol is depleted, cells stop dividing and enter a quiescent state called the stationary phase. ^45^ Studies have demonstrated that adjusting the pH of the growth medium to basic conditions or removing acetic acid from the growth medium can extend CLS. ^46^ It was also shown that transferring stationary phase yeast cells to water, instead of allowing them to age in a carbon source depleted medium, can extend CLS. ^47^ Additionally, it was also demonstrated that, transferring stationary phase yeast cells to water containing only acetic acid reduce the CLS. These findings suggest that extracellular factors, such as acetic acid and pH of the growth medium, plays a key role in the regulation of CLS.

Both intra and extracellular levels of metabolites play a key role in the modulation of CLS. The key metabolites modulating the CLS include acetic acid, ethanol, glycerol, hydrogen sulfide, trehalose, spermidine, reduced nicotinamide adenine dinucleotide phosphate (NADPH), hydrogen peroxide, amino acids, free fatty acids (FFA), sphingolipids, and diacylglycerol (DAG). However, metabolites such as NADPH, trehalose, sphingolipids, FFA, and DAG can influence CLS only within the cell they were produced. Whereas, metabolites such as glycerol, H _2_O _2_, amino acids, spermidine, H _2_S, acetic acid, and ethanol can influence CLS both within the cell they were generated as well as within other cells in the yeast population. ^47^ A set of ligand-specific protein sensors detect changes in the concentrations of these metabolites during chronological aging at certain time points called early and late check points. In early check points, which exist in diauxic and post-diauxic phases, metabolites such as NADPH, glycerol, H _2_O _2_, amino acids, sphingolipids, and spermidine play essential roles in defining the pace of yeast CLS. In contrast, metabolites such as hydrogen sulfide (H _2_S), acetic acid, FFA, and DAG define the pace of chronological aging only at late checkpoints. ^47^

Several factors have been reported to play a crucial in modulating lifespan in yeast. Oxidative stress that arises due to imbalance between ROS production and cellular antioxidants, is considered as one of the common factors responsible for the reduction in both CLS and RLS. Previous studies also showed that deletion of deletion of antioxidant enzymes can influence the lifespan in yeast. Mutants lacking cytosolic copper zinc superoxide dismutase, Cu-Zn SOD (SOD1) and mitochondrial manganese superoxide dismutase, Mn SOD (SOD2) showed a significant reduction in both CLS and RLS. ^48^^,^^49^ The *sod1Δsod2Δ* double mutant had shorter CLS and the *sod1Δ* mutant showed comparatively longer CLS than *sod2Δ*. ^48^ The deletion of catalases did not affect the lifespan. ^49^ Interestingly, the overexpression of SOD1 and SOD2 increased CLS. ^47^ Despite several similarities and differences between CLS and RLS, these two aging paradigms are interrelated as RLS decreases in chronologically aged cells. ^50^ Additionally, both CLS and RLS are regulated by nutrient sensing signaling pathways. ^51^^,^^52^

## Conserved nutrient sensing aging regulatory pathways in budding yeast

**Figure 2.**
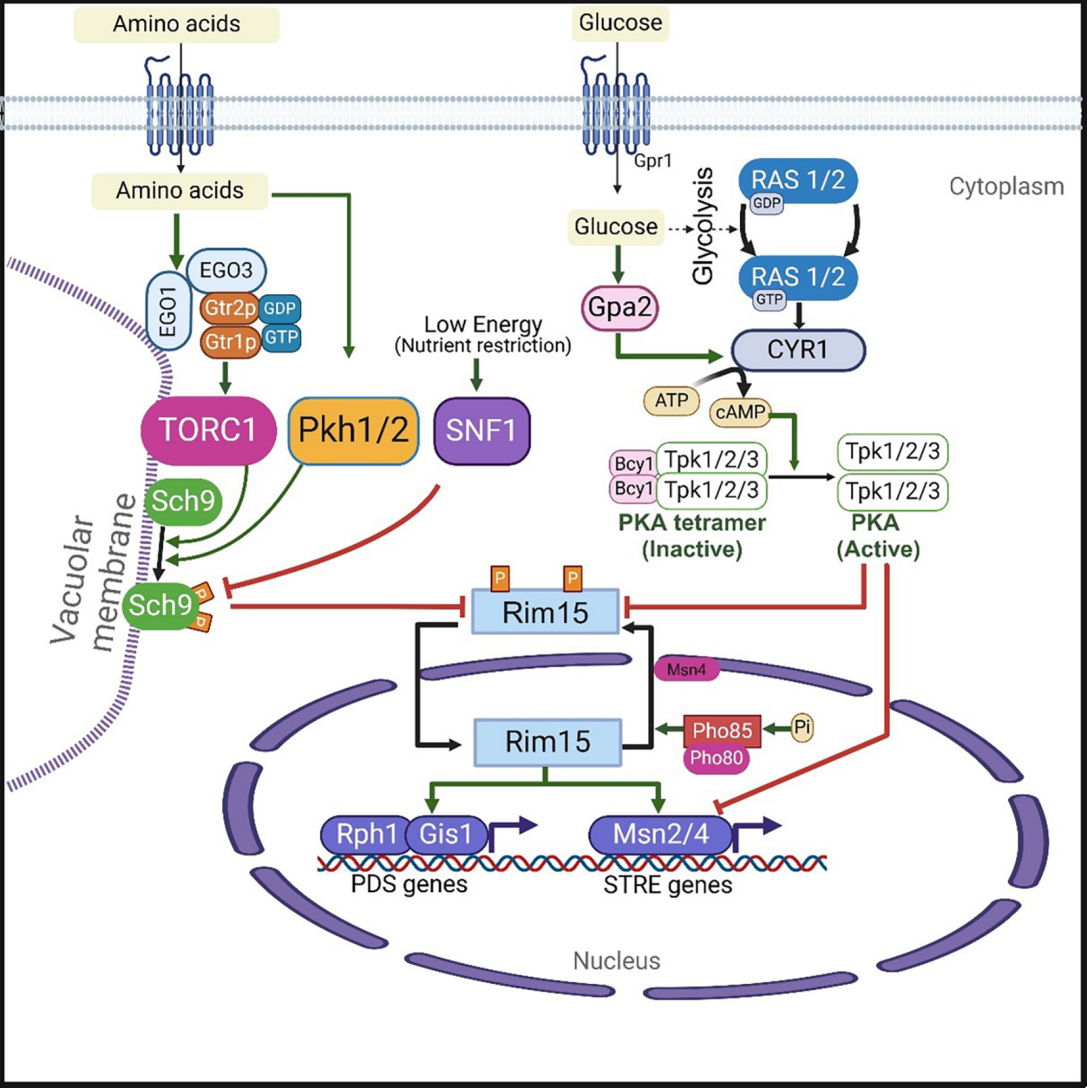
The major nutrient sensing signaling pathways in yeast *Saccharomyces cerevisiae*. Glucose activates adenylate cyclase (CYR1) via stimulation of RAS on one hand and via G protein-coupled receptors (GPCR) system of Gpr1 and Gpa2 on the other hand. The activated adenylated cyclase cause upsurge in the levels of cellular cAMP. Thus, GPR1 and GPA2 are essential for a glucose-dependent rise in the levels of cellular cAMP. Increased intracellular cAMP levels in turn activate protein kinas A (PKA) that exists as a tetramer of two regulatory subunits (Bcy1) and two catalytic subunits (Tpk1, Tpk2 or Tpk3). Binding of cAMP to Byc1 leads to the dissociation of the tetrameric complex resulting in the activation of the catalytic subunits of PKA. On the other hand, amino acids induced activation of TOR1 is mediated by a heterodimer formed from Gtr1p (orthologue of mammalian Rag A/B GTPase) and Gtr2p (ortholog of mammalian Rag C/D GTPase). The Gtr1p GTP - Gtr2p GDP heterodimer interacts with Ego1 (or Gse2) (an ortholog of the mammalian protein MP1), and Ego3 (or Gse1) (an ortholog of the mammalian protein p14), and forms an EGO/GSE complex. This EGOC is localized to the vacuolar membrane via Ego1 subunit. The GTP bound Gtr1p of EGO/GSE complex activates TORC1 which in turn activates Sch9 via phosphorylation. TORC1 can inhibit SNF1 which acts as energy sensor. Both SNF1 and PKH1/2 can influence the activity of Sch9. Both the nutrient signaling pathways RAS-AC-PKA and TORC1-Sch9 converge at Rim15 protein kinase. The activated protein kinases PKA and Sch9 (of RAS-AC-PKA and TORC1-SCH9 signaling pathways) phosphorylates thereby inhibits Rim15 protein kinase activity resulting in the inhibition of its transport to the nucleus. When both PKA and Sch9 are inactive, Rim15 (unphosphorylated form) gets activated and imported to the nucleus, where it induces the activation of transcription factors such as Gis1, Rph1, and Msn2/4. Both Gis1 and Rph1 promotes expression of genes containing postdiauxic shift (PDS) promoter elements. Whereas MSN2/4 induce the transcription of genes containing stress-responsive (STRE) promoter elements. On the other hand, within the nucleus, Rim15 is also inactivated via phosphorylation by a Pho85 cyclin-dependent kinase (in association with its cyclin partner Pho80). Phosphorylated Rim15 is also exported from nucleus to cytoplasm via the Msn5 receptor protein.

Though budding yeast exhibits different aging paradigms, two major nutrient sensing pro-aging signaling pathways, such as the TOR-Sch9 and the RAS-AC-PKA pathways ( Figure 2), promote aging and early death in both RLS and CLS, thus playing a similar role in both aging paradigms, whereas SIR2 does not. ^43^ In presence of nutrients, these pathways promote yeast cell division and growth, but inhibit the general stress response and autophagy. In contrast, the inhibition or inactivation of these pathways results in the extension of both CLS and RLS in yeast. ^43^

### The Ras/Adenylate Cyclase/protein kinase A (PKA) Pathway

RAS genes encode small monomeric proteins called GTPases, which act as signal transducers in all eukaryotes. These Ras proteins are primarily involved in the modulating key cellular processes, including cell growth, proliferation, metabolism, and oncogenic transformation. ^54^^,^^55^ The yeast genes RAS1 and RAS2 are highly homologous to human ras proto-oncogenes, indicating the evolutionary conservation of the ras gene family. Genetic studies have showed that the RAS2 gene can be functionally interchangeable between yeast and humans. ^56^ Mutations in RAS genes have been shown to improve the oxidative stress resistance and increase survival rates in different model systems including yeast, *C. elegans*, drosophila, and mammalian cell lines (e.g., PC12 cells). ^57^ Chronological aging experiments first identified and characterized that the survival of yeast *ras2Δ* mutant increase by cent percent compared to wild-type yeast. Additionally, *ras2Δ* mutant cells demonstrated augmented resistance to both heat stress and oxidative stress. ^57^

In yeast, Ras 1/2 can directly activate adenylate cyclase (Cyr1), which is encoded by CYR1 gene ( Figure 2). In addition to direct activation by Ras 1or 2, the Cyr1 can also be activated by the G protein-coupled receptor Gpr1. ^58^ These two activation ways for Cyr1 are induced when glucose is present in the growth medium. ^59^ Activated Cyr1, in turn, activates a cyclic adenosine monophosphate (cAMP)-dependent protein kinase A (PKA). In its inactive form, PKA exists as a tetramer of two regulatory subunits (Bcy1) and two catalytic subunits (Tpk1, Tpk2 or Tpk3) ( Figure 2). Although the yeast PKA belongs to a different family of serine/threonine kinase, its two catalytic subunits are 35 to 42% identical to *C. elegans* and human AKT-1/AKT-2. ^60^

The binding of cAMP to the regulatory subunits leads to their dissociation from the complex and activation of the catalytic subunits. ^61^ The activation of PKA is essential for cell growth and proliferation. When glucose is abundant, the Ras/cAMP/PKA pathway gets activated to induce the massive expression of genes that promote growth rate in yeast. ^62^ The activated PKA inhibits Rim15, a PAS-kinase that integrates signals from different nutrient sensing signaling pathways such as TORC1, Sch9, PKA, and Pho80-Pho85 to transcription factors Msn2/4 and Gis1 ( Figure 2). ^53^ The inhibition of Rim15 phosphorylation positively regulates the transcription factors such as Msn2/4 and Gis1. It is assumed that Rim15 also play a role in the regulation of genes involved in the glucose repression and cell cycle arrest. ^53^ In yeast, the transcription factors Msn2/4 and Gis1 bind respectively to the STRE (STress Responsive Element) and the PDS (post-diauxic shift) sequences, thereby activate stress resistance genes including SOD1 SOD2, CTT1, HSPs, and DDR2 ( Figure 2). In contrast, the Ras/cAMP/PKA pathway inactivation leads to enhanced heat stress resistance, by partly activating Msn2 and Msn4, which induce the expression of genes encoding for several heat shock proteins (HSPs), superoxide dismutases (SOD1 and SOD2) catalase ( *CTT1*), and the DNA damage inducible gene *DDR2.* ^60^^,^^63^

While the critical functioning of Msn2 and Msn4 are vital for the extension of CLs in both ras2 and cyr1 mutants, previous studies have also demonstrated the essential role of another transcription factor, Gis1, in enhancing stress resistance and extending CLS under Ras/PKA inactivation. ^64^ Thus, the critical roles of Ras/Adenylate Cyclase/PKA Pathway in the regulation of metabolism, stress resistance, proliferation, and longevity in *S. cerevisiae* are mainly linked to the availability of nutrients. ^63^

It was also reported that mutations in CYR1 gene, as well as mutants with impaired synthesis of cAMP, cause extension of CLS in yeast, ^60^ indicating the potential involvement of PKA as a pro-aging pathway. Research evidences also suggests that the pro-aging role of the Ras/adenylate cyclase/PKA pathway seems to be conserved from yeast to mammals. Studies in mice deficient in p66Shc, a signal transducer that might trigger cell proliferation via Ras activation, demonstrated increased oxidative stress resistance and lived 30% longer than wild type. ^65^ Yan et al. further strengthened the critical role of adenylate cyclase (AC5) in the regulation of mammalian aging. They reported that both myocytes and fibroblasts isolated from AC5 knock out mice showed enhanced oxidative stress resistance compared to control cells. Additionally, tissues from AC5 knock out mice, such as brain, heart, and kidneys, showed elevated levels of MnSOD and these mice lived 30% longer than their control littermates, implying the fundamental role of adenylate cyclase AC5 in regulating mammalian lifespan and oxidative stress resistance. ^66^

Enns et al. (2009) provided evidence for the highly conserved pro-aging role of PKA in mammals though experimental studies in RIIB null mice. In mammals, the cAMP dependent-protein kinase A is composed of two regulatory (RI and RII) subunits and two catalytic (C) subunits. The RI and RII subunits exist as two isoforms called RIα, RIβ, RIIα, and RIIβ. ^67^ The RIIβ subunit is predominantly expressed in brown and white adipose tissue and in brain, tissues involved in the regulation of energy homeostasis. RIIβ null mice showed lean body mass and increased median and maximum lifespan compared to wild type littermates. This further supports the essential role of the PKA pathway in mammalian aging as well. ^68^

### The TOR-Sch9 pathway

In response to nutrients, particularly, amino acids, the target of rapamycin (TOR) pathway stimulates anabolic processes and represses catabolic processes such as autophagy, to promote cell growth. ^69^ Target of rapamycin (TOR), a serine threonine kinase was first identified in the budding yeast *S. cerevisiae.* ^70^ *S. cerevisiae* has two TOR protein kinases, Tor1 and Tor2. TOR forms two structurally and functionally distinct complexes: Tor1 containing TOR complex 1 (TORC1) and Tor2 containing TOR complex 1 (TORC2). ^71^ While TORC1 responds to amino acids, controlling cell growth, protein synthesis, metabolism, autophagy, and aging, TORC2 is implicated in the sphingolipid biogenesis, endocytosis, and actin organization. TORC1 is highly conserved between yeast and mammals. ^72^^,^^73^ It consists of subunits such as, TOR1 or TOR2 (ortholog of mammalian TOR, mTOR), Kog1(ortholog of RAPTOR, regulatory-associated protein of TOR), and Lst8 (ortholog of mLST8, mammalian lethal with SEC13 protein 8). Only TORC1 is sensitive to the drugs rapamycin, but not TORC2. ^74^

The TORC1 pathway is the second major pathway implicated in the regulation of aging in yeast ( Figure 2). In response to amino acids levels, Rag GTPases (members of the Ras superfamily) induce activation of mTORC1 signaling in mammals. ^74^ The yeast Gtr1p and Gtr2p are orthologous to mammalian Rag A/B and Rag C/D GTPases, respectively. The amino acid induced activation of TORC1 in yeast is mediated by Gtr1p-GTP and Gtr2p-GDP heterodimer, which interacts with Ego1 (or Gse2) (an ortholog of the mammalian protein MP1), and Ego3 (or Gse1) (an ortholog of the mammalian protein p14), and forms an EGO/GSE complex ( Figure 2). This EGOC is localized to the vacuolar membrane via Ego1 subunit. The GTP bound Gtr1p of EGO/GSE complex activates TORC1, which in turn activates its downstream Sch9 protein kinase (a homolog of human ribosomal S6 kinase 1, S6K1) by phosphorylation. ^75^

*S. cerevisiae* consists of two functionally redundant protein kinases Pkh1 and Pkh2 (homologs of mammalian PDPK1, phosphoinositide-dependent protein kinase-1), which are involved in maintaining the cell wall. ^76^ Both Pkh 1 and 2 protein kinases can also phosphorylate Sch9 kinase. ^77^ In yeast, when glucose levels are low, another protein kinase called SNF1 (sucrose non-fermenting 1 kinase, a homolog of human AMPK) becomes activated and phosphorylate Sch9 ( Figure 2), promoting yeast adaptation to glucose depletion. Thus, the yeast AMPK, SNF1 acts as a key regulator of energy homeostasis. ^78^

Activation of Tor and Sch9 lead to inactivation of Rim15 kinase and the stress resistance transcription factor Gis1 ( Figure 2), both of which are required for maximum CLS extension. ^43^ Similar to Msn2 and Msn4, Gis1 induces the expression of mitochondrial superoxide dismutase enzyme (MnSOD), which is required for the effect of sch9 deletion on CLS. The deletion of *SCH9* can extend both RLS and CLS of yeast. ^60^^,^^79^^,^^80^ Additionally, the combined deletion of SCH9 along with RAS2 can furthermore extend the yeast CLS compared to SCH9 deletion alone. ^80^ Both CLS and RLS can be extended by the loss or inhibition of TOR1 via inactivating the downstream Sch9. ^52^ Aging yeast is characterized by the elevated levels of ROS. In contrast, reduced levels of ROS were observed in the long-lived mutants deficient in Ras-AC-PKA or Tor-Sch9 signaling. ^34^

During CLS, glucose levels decrease gradually and accumulation of non-fermentable carbon sources such as ethanol and acetic acid takes place. Both ethanol and acetic acid promote the chronological aging in yeast. ^45^ Loss of either TOR1or SCH9 slows down the aging process partly by lowering respiration and stimulating the exhaustion of ethanol and acetic acid and the elevating the levels of glycerol in the nutrient medium. ^81^ Since *S. cerevisiae* cannot ferment either acetic acid or ethanol, their depletion by mutations in *TOR* or *SCH9* can prolong the CLS by creating similar conditions usually caused by dietary restriction. It is important to note that the yeast cells deficient in TOR-Sch9 or Ras-AC-PKA signaling pathways exhibit prolonged CLS during incubation in water or in a medium which is either deficient or composed of low amounts of acetic acid and ethanol. ^53^^,^^82^

## Anti-aging studies of natural products and plant extracts in the budding yeast *S. cerevisiae* as a model

Several genes and genetic pathways involved in aging and lifespan extension have been characterized. Over the last 20 years, researcher have identified anti-aging drugs targeting different age-related pathways/mechanisms, including autophagy inducers, epigenetic regulators, and CR mimetics. ^6^ This suggests that targeting both pro-aging and longevity pathways through anti-aging interventions, particularly natural products, contribute significantly to the discovery of natural anti-aging drugs. Natural compounds, which are readily available in food and generally considered much safer for human consumption, serve as one of the main sources of drug discovery and development. Plant derived natural products have been proposed to promote longevity effects through various mechanisms, including 1) maintaining the redox balance, 2) modulating nutrient sensing signaling pathways including the inhibition of mammalian target of rapamycin (mTOR), Insulin/insulin-like growth factor-1 signaling, and nuclear factor-κB (NF-κB) signaling pathways, as well as the activation of adenosine 5’-monophosphate (AMP)- activated protein kinase (AMPK) and sirtuins and, 3) regulating autophagy/mitophagy and mitohormesis, 4) regulating gut microbiome, 5) regulating lipid metabolism, 6) removing senescent cells, 7) activating stem cells and their regeneration, and 8) reducing telomere shortening. ^83^^,^^84^ It is noteworthy that several natural products (e.g., resveratrol, curcumin, spermidine, and curcumin) that demonstrate anti-aging effects in yeast have also entered clinical trials for their potential anti-aging benefits in humans. In the following sections, we have focused mostly on the natural products derived from plants.

### Natural products/plant extracts vs. CLS

Several studies indicate that various natural products and plant extracts have the potential to extend CLS in yeast. These natural products belong to different phytochemical classes, such as: flavonoids (e.g., quercetin), carotenoids (e.g., astaxanthin), ^85^^,^^86^ sesquiterpenes (e.g., artesunate), ^87^ triterpenoids (e.g., betulinic acid), ^88^ flavanones (e.g., Hesperitin) ^89^ and flavonoid glycosides (e.g., Neohesperidin), ^89^ polyphenols (e.g., chlorogenic acid), ^90^ saponins (e.g., Ginsengoside Rg1), ^91^ lignans (e.g., Magnolol), ^92^ etc. Additionally, crude extracts prepared from different plant species were shown to delay yeast chronological aging. In the following section, we summarize the effects of some natural products and plant extracts on the chronological aging in yeast *S. cerevisiae.* Table 1 lists the natural compounds/plant extracts, their concentrations, phytochemical class, type of growth medium used for CLS experiments, and their anti-aging mechanisms in different *S. cerevisiae* strain backgrounds.

**Table 1.** List of Natural products/plant extracts extending the chronological lifespan (RLS) in yeast.

Natural Products						
Name	Chemical Class	Dose	Yeast Strain	Yeast Growth Medium	Anti-Aging Mechanism	Ref.
Acetyl-L-carnitine	Amino acid derivative	1 mM	CML39-11A & BY4741	SD	• ↓ Mitochondrial fission and apoptotic cell death	^93^
Astaxanthin	Carotenoid	30 μM	BY4741	SD	• ↓ MDA, ROS, 8-hydroxy-2-deoxyguanosine (8-OHdG) levels • ↑ SOD and GSH levels • ↓ Apoptotic markers	^85^^,^^86^
Artesunate	Sesquiterpene lactone	0.1, 0.5, 5, 25, & 50 μM	BY4743	YPD	• ↑ Antioxidant defense (Mn SOD) • ↓ ROS • Mimics the effects of CR	^87^
Betulinic acid	Pentacyclic triterpenoid	30 μM	BY4741	SD	• ↓ LPO & ROS • ↓ Apoptotic markers	^88^
Chlorogenic Acid	Polyphenol	5, 10, & 25 μM	DBY746	SDC	• ↓ ROS, RNS, LPO, PCC, and 8-OHdG • Preserving MMP • ↑ SOD1 and SIR2 mRNA levels	^90^
4-N-furfurylcytosine	Pyrimidine derivative	0.25, 0.5, & 1.0 mM	BY4741	YPD	• ↑ mitochondrial respiration • Improved MMP • ↓ ROS levels • ↓ TORC1-Sch9 signaling	^97^
Galactan	Polysaccharide	300 μg/mL	BY4741	SD	• ↓ ROS and MDA levels • ↑ SOD activity • ↓ Apoptotic cell death	^98^
Ginsengoside Rg1	Triterpene saponin	180 g/mL	BY4742	YPD	• ↑ Antioxidant stress response • ↓ ROS levels and apoptosis • ↑ Mitochondrial bioenergetics and glycolytic enzymes	^91^
Glutamic acid and methionine	Amino acids	0.2-fold to 5-fold of normal conc.	BY4742	SD	• Low methionine/glutamic acid ratio in the media increased CLS • High methionine/glutamic acid ratio decreased CLS	^99^
Hesperitin	Flavonone	0.1, 1, 10, & 100 μM	BY4742	SD	• ↓ ROS	^89^
Lithocholic acid	Bile acid	50 μM	BY4742	YPD	• Modulation of carbohydrate and lipid metabolism, mitochondrial structure, and function, liponecrotic and apoptotic cell death • Modulating the expression of stress response transcription factors (e.g., Aft1p, Hog1p, Msn2/4p, Rtg1p- Rtg3p, Sfp1p, Skn7p, and Yap1p)	^100^^“^^103^
Magnolol	lignan	20 μM	BY4741	SCM	• Regulation of SOD1 and CTA1 • Enhanced oxidative stress resistance	^92^
Morusin and mulberrin	Prenylated flavonoids	60 μM	BY4742	SD	• ↑ Oxidative stress response and • ↓ Mutation rate • via targeting the SCH9	^104^
Myricetin	Flavonoid	300 μM	BY4741	SCM	• ↓ ROS and Protein carbonyl levels • ↑ Oxidative stress resistance	^107^
Neohesperidin	Flavanone glycoside	0.1, 1, 10, & 100 μM	BY4742	SD medium	• ↓ ROS	^89^
Quercetin	Flavonoid	300 μM	BY4741	SCM	• ↓ ROS • ↑ Oxidative stress resistance • ↑ Acetic acid stress resistance • ↓ Apoptotic markers	^108^^,^^109^
Spermidine	Polyamine	1 mM	BY4741	SCM	• ↑ Deacetylation of histone H3 by inhibiting histone acetyltransferases • ↓ Oxidative stress and necrosis • ↑ Autophagy via inducing • ↑ mRNA levels of ATG1, ATG5, ATG7, and ATG8	^110^
Sesquiterpene glucosides	Sesquiterpene glucosides	7.5& 10 μM	K6001	YPGal	↑ anti-oxidative stress response	^123^
Tanshinones	Abietane diterpene	20 nM to 5 μM	BY4742	SD medium	• Modulating protein kinases such as Tor1, Sch9, and Gcn2	^111^

Plant Extracts						
Name	Plant Source	Dose	Yeast Strain	Yeast Growth Medium	Anti-Aging Mechanism	Ref.
Almond extracts	Plant extracts	1 mg/mL	DBY746	SDC	• ↓ ROS, RNS, LPO, PCC, & 8-hydroxy-2-deoxyguanosine • Preserving MMP • ↑ SOD1 and SIR2 mRNA levels	^90^
Coffee	Seeds	0.6 mg/mL	BY4741	SDC	• ↓ ROS levels • ↓ Metabolic activity • ↓ Double DNA-strand breaks	^112^
*Polyalthia longifolia*	Extract	1 mg/mL	BY611	YPD	• ↓ ROS levels • ↑ GSH levels • ↑ mRNA expression of *SOD* and *SIR2* genes	^113^
*Nelumbo nucifera*	Stamen	0.5 mg/mL	DBY746	SDC	• ↑ Antioxidant status (both enzymatic activity and gene expression levels of SOD1 and SIRT2)	^124^
Rice bran extract	Extract	0.001, 0.01, 0.1, 1 mg/mL	BY4742	SD	• ↓ ROS levels • maintain the plasma membrane integrity • through the modulation of TOR1 and SIR2-dependent pathway	^116^
*Cimicifuga racemosa*	Root & rhizome	0.5% (w/v)	BY4742	SD	• ↓ Pro-aging TORC1 pathway • ↑Anti-aging SNF1 pathway	^118^
*Valeriana officinalis*	Root	0.5% (w/v)	BY4742	SD	• ↓ Pro-aging PKA pathway	^118^
*Ginkgo biloba*	Leaf	0.3% (w/v)	BY4742	SD	• ↓ inhibitory action of PKA on SNF1	^118^
*Apium graveolens*	Seed	0.1% (w/v)	BY4742	SD	• Stimulating Rim 15 protein kinase	^118^
*Salix alba*	Bark	0.1% (w/v)	BY4742	SD	• ↓ Pro-aging Sch9	^118^
*Salix alba*	Bark	0.1% (w/v)	BY4742	SD	• Remodeling of intracellular and mitochondrial lipid metabolism by • ↓ Intracellular levels of free fatty acids, which results in the postponement of an age-related onset of liponecrotic cell death • ↓ Triacylglycerols and to increase the concentrations of glycerophospholipids within the endoplasmic reticulum membrane, which results in the activation of the unfolded protein response system in the ER, which then decelerates an age-related decline in protein and lipid homeostasis • Changing lipid concentrations in the mitochondrial membranes.	^121^
*Manihot esculenta* & *Wodyetia bifurcata*	Leaf	100 μg/mL	1783	SCM	• ↑ Oxidative stress response	^122^

YPD (1% yeast extract, 2% peptone, 2% dextrose); YPGal (1% yeast extract, 2% peptone, 2% Galactose); YPGly (1% yeast extract, 2% peptone, 3% Galactose); SCM (synthetic complete medium); SD (Synthetic defined/synthetic minimal) medium; SDC (synthetic complete dextrose) medium. Symbols ‘↑’ and ‘↓’ indicate up-regulation/increase and down-regulation/decrease, respectively.

### Acetyl L-carnitine

Acetyl-L-carnitine (ALC) is an endogenous molecule synthesized in the body from L-carnitine. ALC plays a key role in the energy metabolism and it has been reported to exhibit a plethora of pharmacological properties, including neuroprotective activities. It has also been used as a dietary supplement due to its potent therapeutic effects. Researchers have shown the anti-apoptotic and anti-aging ability of ALC in yeast model. ALC was found to inhibit mitochondrial fission and subsequently improve mitochondrial functioning. Yeast gene MCA1 encodes a Ca+2-dependent protease which plays a key role in the regulation of apoptosis. It was found that the mitoprotective effects of ALC were mediated through the yeast metacaspase (Yca1) and thus to its anti-apoptotic activity. ALC was also shown to extend the CLS of yeast cells. ^93^

### Astaxanthin

Astaxanthin is a carotenoid compound with superior antioxidant ability than many other natural antioxidant molecules. Sudarshan *et al.* conducted a study to test the antioxidant, anti-apoptotic and anti-aging properties of astaxanthin using yeast model. Astaxanthin treatment prevented oxidative stress-induced surge in MDA and ROS levels and reduction in superoxide dismutase and glutathione levels, thereby increasing the percentage viability of antioxidant gene-deleted yeast mutants by 20-40%. In addition, astaxanthin also prevented the apoptosis of aged cells and subsequently increased the viability of yeast cells during the CLS. Astaxanthin’s antioxidant and anti-apoptotic effects are the possible reason for its anti-aging activity. ^85^ It was also suggested that anti-apoptotic effects of astaxanthin are also due to its ability to prevent nuclear fragmentation and chromatin condensation in yeast cells. ^86^ Sudarshan *et al*. also found that astaxanthin improved oxidative stress resistance and increased the viability of yeast DNA damage repair gene-deleted mutant cells. Astaxanthin was shown to prevent the accumulation of endogenous DNA damage marker (8-hydroxy-2-deoxyguanosine) levels in yeast cells. The anti-aging effects of astaxanthin were also suggested to be due to its ability to inhibit the accumulation of mutation during chronological aging of DNA damage repair gene-deleted mutant yeast cells. ^86^

### Artesunate

Artesunate is a semi-synthetic derivative of the antimalarial drug artemisinin. Previous studies have shown that artesunate exerts anti-aging effects similar to caloric restriction, indicating the CR mimetic effects of artesunate. From the whole-transcriptome profile analysis studies, it was revealed that artesunate mimics CR-triggered nitric oxide to induce antioxidant defense systems, thereby preventing ROS accumulation and mitigating oxidative stress, subsequently extending yeast lifespan. ^87^

### Betulinic acid

Betulinic acid (BA) is a pentacyclic triterpenoid present in some plant species. A recent study reported that betulinic acid extends the CLS of yeast cells by mitigating oxidative stress-induced apoptosis. The anti-aging effects of betulinic acid were found to be mediated by the induction of genes associated with heat shock stress response and autophagy. ^88^

### Citrus flavonoids

Flavonoids have been reported to exert a plethora of pharmacological activities including antioxidant, anti-inflammatory, anti-cancer, anti-neurodegenerative, anti-aging, among others. Researchers have investigated the anti-aging effects of citrus flavonoids including naringin, hesperedin, hesperitin, and neohesperidin on the chronological aging of yeast. Among the tested flavonoids, neohesperidin significantly prevented accumulation of ROS and extended the chronological life span of yeast in a concentration-dependent manner. ^89^ In another study, treatment with hesperidin significantly induced the expression levels of SOD and sirtuin2 (SIR2) and prevented ROS accumulation in yeast. In yeast, *UTH1* gene encodes Uth1p, which is activated by oxidative stress, senescence, and TOR-dependent autophagy; all these events subsequently lead to cell death in yeast. Pretreatment with hesperidin, but not its aglycon hesperetin, significantly inhibited the expression of *UTH1* gene, thereby extending yeast lifespan. ^94^

### 4-N-furfurylcytosine

Previous studies have demonstrated that purine and pyrimidine derivates can exhibit promising health promoting effects. ^95^^,^^96^ Pawelczak *et al.* investigated the antiaging effects of 4-N-Furfurylcytosine (FC), a cytosine derivative using model *S. cerevisiae.* They found that treatment with FC increased the percentage viability of yeast cells during CLS in a concentration-dependent manner. In addition, treatment with FC boosted the mitochondrial activity and reduced intracellular levels of ROS. It was also demonstrated that FC could limit TORC1 signaling in yeast. This points to the fact that FC’s anti-aging activity is through the inhibition of TOR signaling pathway. ^97^

### Galactan exopolysaccharide

A recent study using yeast models investigated the antioxidant and anti-aging properties of galactan exopolysaccharide isolated from a gram-positive *Weissella confusa.* Galactan exopolysaccharide was shown to prevent the oxidative stress induced rises in ROS levels and enhance the viability of yeast cells exposed to hydrogen peroxide as an oxidant used in this study. In addition, galactan exopolysaccharide treatment significantly increased the viability of both wild type and antioxidant gene-deleted mutant ( *sod2∆*). The study suggested that the antioxidant potential of the galactan exopolysaccharide could be the possible reason for its anti-aging activity. ^98^

### Ginsengosides

Ginsenosides are major pharmacological compounds that are unique to the plant species *Panax ginseng* C. A. Meyer (ginseng). Ginsenosides have been demonstrated to extend the life span of different model organism. In a recent study, researchers treated yeast cells with ginsenoside Rg1 and found an augmented antioxidant stress response and concomitant reduction in ROS levels and apoptosis, thereby increasing the viability during aging in yeast model. It was also demonstrated that ginsenoside Rg1 treatment resulted in increased mitochondrial bioenergetics and glycolytic enzymes, thereby improving metabolic homeostasis and delaying aging in *S. cerevisiae.* ^91^

### Glutamic acid and methionine

A study ^43^ reported that the composition of amino acids in aging media can affect the chronological life span of yeast cells. The presence of non-essential amino acids methionine and glutamic acid have been reported to greatly influence the survival rate of yeast cells during aging. Precisely, low levels of methionine and high levels of glutamic acid in the yeast nutrient media led to extended lifespan in yeast model. In contrast, increasing levels of methionine and reducing levels of glutamic acid caused a decrease in yeast life span. Therefore, it can be concluded that amino acid composition is a critical factor for controlling yeast aging. ^99^

### Lithocholic acid

Previous studies have reported that lithocholic acid, a bile acid, can increase yeast cell survival during chronological aging. ^100^^–^^103^ Interestingly, lithocholioc acid was also shown to significantly enhance the survival rate of yeast especially under caloric restriction conditions. Lithocholic acid treatment was shown to modulate several cellular pathways, including carbohydrate and lipid metabolism, mitochondrial structure and function, liponecrotic and apoptotic cell death of yeast during chronological aging. ^100^ It is important to note that lithocholic acid’s anti-aging effects under caloric restriction are found to be prominent only when this compound is added to the growth medium either at logarithmic/diauxic and early stationary stages of yeast aging. Addition of lithocholic acid either at logarithmic/diauxic and early stationary stages induced the activation of several longevity related cellular processes which ultimately triggering the enhanced yeast cell survival during aging. ^101^ Beach *et al.* also demonstrated that lithocholic acid exerts its anti-aging effects through the modulation of the expression of different transcription factors including Aft1p, Hog1p, Msn2/4p, Rtg1p- Rtg3p, Sfp1p, Skn7p, and Yap1p. Each of these transcription factors in turn alter the levels of several intra and extra mitochondrial proteome, and subsequent maintenance of mitochondrial function. Altogether, Beach *et al.* suggested that lithocholic acid’ anti-aging effects are mainly through the modulation of aging-related transcriptional landscape. ^102^ Also, it was discovered that lithocholic bile acid accumulates in mitochondria, and alters the mitochondrial membrane lipidome which is crucial for the restoration of mitochondrial proteome, subsequently an improved mitochondrial function and increased chronological aging by lithocholic acid. ^103^

### Magnolol

In our previous study, we reported the anti-aging effects of magnolol, a natural polyphenol. Magnolol enhanced the stress resistance of yeast cells exposed to hydrogen peroxide, an oxidizing agent. In addition, magnolol increased the viability of the short-lived yeast mutant *sod1∆*, which lacks the antioxidant enzyme superoxide dismutase. Our study suggested that the anti-aging effects of magnolol occur mainly through the modulation of oxidative stress during yeast chronological aging. ^92^

### Morusin and mulberrin

Mulberry leaves are rich in flavonoids such as morusin and mulberrin, and these have been demonstrated to enhance the survival rate during yeast chronological aging. It was also found that morusin and mulberrin exert their anti-aging effects via targeting the SCH9, a major target of TORC1 in budding yeast *S. cerevisiae.* ^104^ Budding yeast Sch9 is the major substrate of the TORC1. Yeast Sch9 functions analogously to S6K1, the mammalian TORC1 substrate. Yeast TORC1 phosphorylates about six amino acid residues of Sch9, indicating that the TORC1-dependent phosphorylation of Sch9 is essential for its activity. ^105^ Activated Sch9 plays a key role in the regulation of several cellular processes including protein synthesis (via modulating ribosome synthesis and translation initiation), cell cycle, and aging. Deletion of SCH9 causes growth defects such as reduction in cell size and growth rate; however, deletion of SCH9 leads to enhanced temperature tolerance as well chronological and replica life spans in budding yeast. The longevity-extending effects due to SCH9 deletion are explained by the augmented oxidative stress response and reduction in age-associated mutation rate. ^106^

### Myricetin

In another study, using budding yeast as a model, researchers investigated antioxidant and anti-aging effects of myricetin, a polyphenolic flavonoid compound which is composed of a pyrogallol ring in the B ring of its structure. Myricetin’s antioxidant effects are attributed to the presence of a hydroxyl group in its B ring. Treatment with myricetin reduced the levels of ROS and protein carbonyl content, thereby enhancing the oxidative stress resistance of yeast cells exposed to hydrogen peroxide. Myricetin was found to inhibit H _2_O _2_-induced glutathione oxidation, but did not enhance endogenous antioxidant enzymatic activity in yeast. Additionally, myricetin treatment significantly prevented age associated oxidative stress and extended the CLS of yeast mutant lacking mitochondrial superoxide dis-mutase ( *sod2Δ*). ^107^

### Quercetin

It is noteworthy that several of the yeast genes, such as, PEP4 and TEL1, share homology with human genes of diseases relevance, such as CTSD and ATM, respectively. ^108^^,^^109^ In a previous study, we demonstrated the protective effects of quercetin on *S. cerevisiae pep4Δ* mutant that lack the *PEP4* gene encoding a vacuolar endopeptidase proteinase A. This vacuolar proteinase A is a homolog of the human cathepsin D (encoded by CTSD), which plays a key role in the normal development and maintenance of neurons in the central nervous system. Particularly, it is essential for the degradation of proteins linked to several neurodegenerative diseases such as Parkinson’s disease, Huntington disease, neuronal ceroid lipofuscinosis, and Alzheimer’s disease. ^108^ Yeast *pep4Δ* cells are found to be highly sensitive to oxidative and apoptotic stressors. However, in our study, we demonstrated that quercetin treatment reduced ROS levels and apoptotic markers, thereby increasing the percentage viability of yeast *pep4Δ* cells during chronological aging. ^108^

The mammalian serine/threonine protein kinase ATM plays an important role in DNA damage sensitivity, cell cycle checkpoint deficiency, cancer incidence and telomere length maintenance. Mutation in ATM gene is linked to elevated oxidative damage, premature aging, and apoptosis. In another study, we also investigated the protective effects of quercetin on the sensitivity of *S. cerevisiae tel1Δ* cells lacking Tel1p, which is a homolog of the human *ATM* (Ataxia Telangiectasia Mutated) gene mutation. Our study results showed that quercetin treatment prevented ROS accumulation and thereby enhanced the stress resistance of *tel1Δ* cells exposed to a variety of oxidizing agents. Furthermore, treatment with quercetin prevented apoptotic death of yeast *tel1Δ* cells and increased cell viability during chronological aging. ^109^

### Spermidine

Spermidine has been reported to inhibit histone acetyltransferases and subsequent deacetylation of histone H3, thereby preventing oxidative stress and necrosis in yeast aging. On the contrary, reduction of endogenous polyamines caused hyperacetylation, accumulation of ROS, necrotic cell death and a diminished life span. It is important to note that treatment with spermidine causes alteration in acetylation status of the chromatin, resulting in a substantial increase in the autophagy in different model organisms, including yeast. This suggests that spermidine’s anti-aging effects are mediated through its ability to stimulate the autophagy pathway. ^110^

### Tanshinones

Wu *et al*. found that the dried roots of *Salvia miltiorrhiza* Bunge have substantial longevity extending effects. They reported that tanshinones ( *e.g.*, cryptotanshinone, tanshinone I, and tanshinone IIa) are pharmacologically active components present in the roots of *S. miltiorrhiza.* Precisely, cryptotanshinone has been reported to extend the CLS of yeast wild type as well as yeast mutant lacking mitochondrial superoxide dismutase ( *sod2Δ*). Their study suggests that the cryptotanshinone-induced anti-aging effects are modulated through the involvement of nutrient-sensing protein kinases such as Tor1, Sch9, and Gcn2. ^111^

### Almond extracts

Almond ( *Prunus dulcis* (Mill.) D.A. Webb) is a major nut crop worldwide. Multiple health benefits associated with the consumption of almonds are due to the phenolic-rich almond skin. Previous studies have found that pre-treatment with almond skin extract and chlorogenic acid significantly extended the lifespan of yeast. Both almond extract and chlorogenic acid improved the mitochondrial function during chronological aging of yeast cells by reducing the accumulation of free radicals (including ROS and RNS) and preserving mitochondrial membrane potential (MMP). Furthermore, treatment with almond extract and chlorogenic acid augmented the oxidative stress response by inducing the expression levels of *SIR2* and *SOD1* genes and decreasing the endogenous levels of lipid peroxides, protein carbonyls and 8-hydroxy-2-deoxyguanosine (8-OHdG) in yeast cells. Altogether, it can be suggested that the longevity-extending effects of almond extract are ascribed to its ability to ameliorate oxidative stress in yeast cells. ^90^

### Coffee

Czachor, J., *et al.* demonstrated the lifespan-extending effects of coffee infusions in *S. cerevisiae.* Coffee, especially the *Coffea robusta* type, was found to exhibit superior antioxidant effects than the *Coffea arabica* type, thereby protecting cells from ROS-induced DNA damage and additionally improving the metabolic activity in yeast cells. overall, it can be suggested that coffee exhibits health benefits mainly through the reduction of ROS accumulation and the enhancement of metabolic activity. ^112^

### Polyalthia longifolia

*Polyalthia longifolia* is a polyphenol-rich traditional medicinal plant that has long been used for its rejuvenation capacity. *P. longifolia* has been reported to exhibit potent pharmacological activities including antioxidant and hepatoprotective activities. A previous study, ^63^ tested the anti-aging activity of methanolic leaf extract of *P. longifolia* using yeast CLS model and found that the methanolic leaf extract enhanced the viability of yeast cells and extended the CLS of yeast. It was reported that methanolic leaf extract significantly prevented the accumulation of H _2_O _2_-induced ROS levels and increased GSH levels. Furthermore, treatment with methanolic leaf extract strikingly induced the expression levels of *SOD* and *SIRT1* genes. Overall, it can be suggested that *P. longifolia* exerts its anti-aging effects through the modulation of oxidative stress response and *SIRT1* gene. ^113^ It was also reported that methanolic leaf extract of *P. longifolia* extended the RLS of yeast via ameliorating the apoptotic features of replicatively ageing yeast cells. ^114^

### Rice bran extract

Pigmented rice is the functional food in many countries including India, China, and Japan, and it is the richest source of polyphenols. ^115^ It has been reported that red rice bran extract can prevent the accumulation of ROS levels, maintain plasma membrane integrity and extend the CLS of yeast cells. The anti-aging mechanism of action of rice bran extract is through the modulation of the TOR1 and SIR2-dependent pathways. ^116^

### Sacred lotus stamen extract

The stamen of lotus ( *Nelumbo nucifera*) is rich in flavonoids and has long been widely used in Indian traditional medicine. Tungmunnithum *et al.* reported that treatment with lotus stamen extract significantly enhanced the antioxidant status (both enzymatic activity and gene expression levels of SOD1 and SIRT2) and extended the CLS of yeast cells. Interestingly, the longevity extending effects of lotus stamen extract were reported to be superior to the resveratrol treatment. ^117^

Previous studies also demonstrated that a variety of plant extracts can extend the CLS in yeast by modulating both pro-aging and anti-aging protein kinases. Plants extracts of *Apium graveolens* and *Cimicifuga racemosa* have been shown to stimulate the anti-aging protein kinases Rim15 and SNF1, respectively. ^118^ Both *Valeriana officinalis* and *Ginkgo biloba* extracts were shown to exert inhibitory action on the pro-aging PKA pathway, whereas, the anti-chronological aging effects of *Cimicifuga racemosa* and *Salix alba* were shown to be modulated via inhibitory action on the pro-aging kinases Sch9 and TORC1, respectively. ^118^

The anti-aging potential of these plant extracts were also attributed to their ability to enhance hormetic stress response in yeast. Precisely, these extracts were shown to increase mitochondrial respiration and membrane potential, decrease or increase ROS, enhance resistance to both thermal and oxidative stress, and mitigate oxidative damage to protein, lipids, and DNA. ^119^^,^^120^ Additionally, *Salix alba* extract was demonstrated to exhibit anti-chronological aging effects via remodeling of both intracellular and mitochondrial lipid metabolism, particularly, by decreasing intracellular free fatty acid levels, resulting in delay in the age-related liponecrotic yeast cell death. ^121^ Kwong, M.M.Y. et al. (2021) screened for the anti-aging potential of 222 plant extracts and their findings revealed that two plant extracts namely *Manihot esculenta* and *Wodyetia bifurcata* could extend the CLS via inducing the oxidative stress response pathways in yeast. ^122^

### Natural products/plant extracts vs. RLS

Researcher also demonstrated the potential of natural products and plant extracts to extend the RLS in yeast. These natural products belong to different phytochemical classes, such as secoiridoid glycoside (e.g., amarogentin), ^125^ flavonone glycoside (e.g., hesperidin), ^94^ triterpenoid glycoside (e.g., cucurbitane glycoside), ^126^ ergosterol derivatives (e.g. ganodermasides A and B), ^127^ phospholipid (e.g., lysophospholipid), ^128^ flavonoid (e.g., phloridzin), ^129^ stilbenoid (e.g., reservatrol), ^130^ and polyphenolic glycosides (e.g., parishin). ^131^ Others also demonstrated that crude extracts prepared from different plant species including *Polyalthia longifolia,* ^114^ *Psoralea corylifolia,* ^132^ and *Pterocarpus marsupium* ^133^ extended RLS in yeast. In the following section, we summarize the effects of some natural products and plant extracts on the replicative aging in yeast *S. cerevisiae.* Table 2 lists the natural compounds/plant extracts, their concentrations, phytochemical class, type of growth medium used for RLS experiments, and their anti-aging mechanisms in different *S. cerevisiae* strain backgrounds.

**Table 2.** List of Natural products/plant extracts extending the replicative lifespan (RLS).

Natural Products						
Name	Chemical Class	Dose	Yeast Strain	Yeast Growth Medium	Anti-Aging Mechanism	Ref.
Amarogentin	Secoiridoid glycoside	1, 3 and 10 μM	K6001	YPGal	• ↑ SOD, CAT, and GPx activities • ↑ mRNA levels of SOD1 and SOD2, CAT, and GPx activities	^125^
Hesperidin	Flavanone glycoside	5, 10, and 50 μM	K6001	YPGal	• ↑ SOD1, UTH1, and SIR2 mRNA levels • ↓ ROS	^94^
Cucurbitane glycoside	Triterpene glycoside	1, 3, and μM	K6001	YPGal	• ↓ ROS levels • ↓ mRNA levels of UTH1 and SKN7 • ↑ mRNA levels of SOD1 and SOD2	^126^
Copper sulfate	Inorganic compound	62 μM	W303-1A, S288c, BY4742, SP-22	YPGly	• ↑ Respiratory metabolism • Activation of stress response genes • Improved mitochondrial function	^137^^,^^138^
Ferric chloride	Inorganic compound	1 mM	BY4742	YPGly	• Activation of multicopper oxidase enzyme Fet3p • ↓ ROS levels • ↑ mRNA levels of genes related to the mitochondrial metabolism (e.g., TCA cycle and electron transport chain) • Elevated levels of ATP • Amelioration of mitochondrial energy metabolism	^137^^–^^139^
Ganodermasides A and B	Ergosterol derivatives	1, 10, and 100 μM	K6001	YPGal	• via modulating the expression of UTH gene	^127^
Isonicotinamide	Amide form of isonicotinic acid	25 mM	BY4741	SCM	• ↑ Intracellular levels of NAD+ via NAD+ salvage pathway • ↑ Sir2 activation, subsequent increase in silencing at the rDNA locus	^144^
Lysophosphatidic acid	Bioactive phospholipid	10 and 30 μM	K6001	YPGal	• ↑ oxidative stress resistance • amelioration of antioxidant status and the genes of *UTH1*, *SKN7*, and *SOD*	^128^
Nicotinamide Riboside	Form of vitamin B3	10 μM	BY4742	YPD	• ↑ NAD+ levels via activation of both Nrk1-dependent and Nrk1-independent pathways • ↑ Sir2-dependent gene silencing	^142^
Parishin	Polyphenolic glycoside	3, 10, and 30 μM	K6001	SCGal	• ↑ expression of levels of SIR2 and SOD activity, • ↓ ROS and lipid peroxidation • ↓ mRNA levels of TOR signaling-related genes (e.g., TORC1, RPS26A, and RPL9A	^131^
Phloridzin	Flavonoid	3, 10, and 30 μM	K6001	YPGal	• ↑ mRNA levels of SOD1, SOD2, and SIRT1	^129^
Rapamycin	Macrolide	200 ng/ml	BY4741	SCM	• ↓ TORC1 complex • Stabilization of rDNA locus via increased association of Sir2 with ribosomal DNA (rdna) • ↓ Extrachromosomal rDNA circles	^148^
Resveratrol	stilbenoid	30 μM	W303-1a	SCM	• ↓ Number of senescent cells with fragmented mitochondria • ↑ Mitochondrial DNA content • ↑ mRNA levels of DNM1 and FZO1 genes • ↓ROS	^130^
Sesquiterpene glucosides	Sesquiterpene glucosides	7.5 and 10	K6001	YPGal	• ↑ anti-oxidative stress response	^123^

Plant Extracts						
Name	Plant Source	Dose	Yeast Strain	Yeast Growth Medium	Anti-Aging Mechanism	Ref.
*Psoralea corylifolia*	Extract	10 μg/mL	MEP strain ZHY1	YPD	• Via modulating Tor1 • Sir2-independent pathway	^132^
*Polyalthia longifolia*	Extract	1 mg/mL	BY611	YPD	• Amelioration of apoptotic features	^114^
*Pterocarpus marsupium*	Extract	100 μg/mL	BY4742	YPD	• Anti-aging effects might involve alterations in cellular processes related to aging	^133^

YPD (1% yeast extract, 2% peptone, 2% dextrose); YPGal (1% yeast extract, 2% peptone, 2% Galactose); YPGly (1% yeast extract, 2% peptone, 3% Galactose); SCM (synthetic complete medium); SD (Synthetic defined/synthetic minimal) medium; SDC (synthetic complete dextrose) medium. Symbols ‘↑’ and ‘↓’ indicate up-regulation/increase and down-regulation/decrease, respectively.

### Amarogentin

Disasa *et al.* isolated a secoiridoid glycoside amarogentin from a Chinese traditional medicinal plant, *Gentiana rigescens* Franch. Amarogentin was reported to enhance both the enzymatic activities and expression levels of superoxide dismutase (SOD), catalase (CAT), and glutathione peroxidase (GPx), thereby improving the viability of yeast cells exposed to oxidative stress. Furthermore, treatment with amarogentin resulted in an enhanced RLS of yeast wild type; however, this compound was not shown to extend the lifespan of yeast mutants *sod1Δ*, *sod2Δ*, *uthΔ*, and *skn7Δ*, suggesting that the anti-aging effects of amarogentin are mainly through the regulation of antioxidative stress as well as by the regulation of *UTH1*, *SKN7*, *SOD1*, and yeast *SOD2* gene expression. ^125^

### Cucurbitacins

Cucurbitacins are a class of tetracyclic triterpenoids that are abundant in plants belonging to the family Cucurbitaceae. Cucurbitacins have been reported to exhibit strong anticancer activity, and are divided into 12 classes from A to T with over 200 derivatives. ^134^ Cucurbitacin B is the most abundant and active member of the cucurbitacins. ^135^ Researchers have discovered that cucurbitacin B can extend both replicative and chronological life spans in yeast. Treatment with cucurbitacin B enhanced the lifespan of yeast by promoting the expression of *ATG32.* Cucurbitacin B could not increase the lifespan of yeast mutants devoid of autophagy related genes ( *ATG2* and *ATG32*), pointing to the fact that the anti-aging effects of cucurbitacin B are mainly through the induction of autophagy in yeast.

It was also demonstrated that cucurbitacin B can ameliorate oxidative stress levels mainly through the induction of superoxidase dismutase activity (via enhanced expression of *SOD1* and *SOD2*) and the prevention of accumulation of oxidative stress markers including ROS and malondialdehyde (MDA). Furthermore, researchers also showed that the anti-aging effects of cucurbitacin B are also mediated through the regulation of expression of other aging-related genes such as *UTH1* as well as *SKN7.* Cucurbitacin B’s anti-aging effects are attributed to its ability to ameliorate oxidative stress and regulate autophagy and age-related genes in yeast. ^136^

Additionally, cucurbitane glycoside isolated from the methanol extracts of *Momordica charantia* L. fruits has been reported to significantly extend the RLS of K6001 budding yeast mainly through suppressing ROS levels and the oxidative stress burden. Treatment with cucurbitane glycoside resulted in a decrease in the expression levels of *UTH1* and *SKN7* and an increase in the expression levels of *SOD1* and *SOD2.* However, cucurbitane glycoside was not able to extend the RLS of the yeast mutants *uth1Δ*, *skn7Δ*, *sod1Δ*, and *sod2Δ*, suggesting that cucurbitane glycoside exerts antiaging effects via antioxidative stress and regulation of yeast *UTH1*, *SKN7*, *SOD1*, and *SOD2* gene expression. ^126^

### Copper and iron supplementation

Previous studies were conducted to demonstrate the effects of supplementation of copper and iron on the yeast RLS. It was found that addition of copper to the growth media increased RLS via the activation of multicopper oxidase enzyme (Fet3p) that works in combination with another enzyme called iron permease (Ftr1p), allowing for high intake of iron into yeast cells. Furthermore, it is important to note that high levels of iron-mediated anti-aging effects are dependent on the multicopper oxidase enzyme. However, the life span-extending effects of both copper and iron occurred in the growth medium supplemented with only glycerol as a carbon source but not glucose.

It was also demonstrated that supplementation of either copper or iron prevented the accumulation of ROS levels, thereby enhancing the replicative life span of yeast mutants lacking antioxidant enzymes. ^137^^,^^138^ The iron-mediated anti-aging effects occur mainly through the enhanced expression of genes related to the mitochondrial metabolism especially tricarboxylic acid (TCA) cycle and electron transport chain, subsequently elevating levels of adenosine triphosphate (ATP), which is essential for increased survival cells during aging. Interestingly, supplementation of iron could also enhance the life span of yeast *Snf1Δ* mutant which lacks SNF1/AMPK protein kinase. Therefore, it can be suggested that the iron’s life span-extending effects occur via amelioration of mitochondrial energy metabolism in yeast. ^139^

### Ganodermasides A and B

Medicinal mushrooms have been reported to exhibit anti-aging activity. In a study, ^38^ researchers isolated two ergosterol derivatives, namely ganodermasides A and B, from the spores of the medicinal mushroom *Ganoderma lucidum.* They showed that both ganodermasides A and B could extend the replicative life span of *S. cerevisiae* through the modulation of the expression of UTH. In yeast, different transcription factors such as Skn7, Yap1, and Mot3 play a crucial role in oxidative stress resistance. It was suggested that upon phosphorylation, these transcription factors get activated, thereby regulating the expression of *UTH1* gene by binding to its upstream promoter region. It was reported that polyphenols’ anti-aging activity occurs through the activation of Skn7 and subsequent increased expression of the *UTH1* gene. ^127^

### Lysophosphatidic acid

In another study, Sun Y *et al.*, isolated lysophosphatidic acid (LA) from the seeds of *Arabidopsis thaliana* and showed that LA augmented oxidative stress resistance, thereby promoting the extension of RLS in yeast. However, LA was not able to extend the replicative lifespan of mutants *uth1Δ*, *skn7Δ*, *sod1Δ*, and *sod2Δ.* This study suggests that LA’s anti-aging effects are possibly through the amelioration of antioxidant status of yeast cells and the genes of *UTH1*, *SKN7*, and *SOD* may also be involved in the action. ^128^

### Nicotinamide adenine dinucleotide (NAD+) precursors

Alterations in the biosynthesis and regulation of nicotinamide adenine dinucleotide (NAD+) plays a crucial role in the progression of aging as well as pathophysiology of several age-related chronic diseases. ^140^ NAD + plays a crucial role in several metabolic processes in the cells. Most importantly, NAD+ functions as a co-factor in redox reactions and it is reduced to NADH in many metabolic pathways including glycolysis, the citric acid cycle, and fatty acid metabolism. Adequate intracellular levels of NAD+ are maintained through the combined action of NAD(+) biosynthesis and salvage pathways.

Several key cellular enzymes including sirtuin protein deacetylases and poly-ADP-ribose polymerases (PARPs) are NAD+ dependent, and use NAD+ as a substrate in many cellular processes, including aging. Aging is associated with a reduction in NAD+ levels, leading to alterations in several metabolic processes that are NAD+-dependent, which in turn leads to the ageing-associated physiological/functional decline. Therefore, supplementation with diets rich in three important NAD+ precursors 1) nicotinamide, 2) nicotinic acid ( *e.g.*, protein rich foods such as cereals, peanuts, meat, fish) and 3) nicotinamide riboside ( *e.g.*, milk, cabbage, cucumber) ameliorate age-related decline in NAD+ levels and help in the extension of active longevity. ^141^

Nicotinamide riboside is a natural product present in milk. ^30^ NAD+ is also required for the proper functioning of Sir2 and extension of RLS in budding yeast. Alterations in NAD+ levels negatively affect the RLS of yeast. In eukaryotes, nicotinamide riboside kinases (Nrk1 and Nrk2) catalyze the phosphorylation of nicotinamide riboside to nicotinamide mononucleotide, the precursor of NAD+. A previous study reported that the supplementation of nicotinamide riboside increased the levels of NAD+ via activation of both Nrk1-dependent pathway and the Urh1/Pnp1/Meu1 pathway (an Nrk1-independent pathway).

Increased NAD+ levels in turn lead to enhanced Sir2-dependent gene silencing, thereby extending RLS in yeast. ^142^ The Sir2 enzyme deacetylates the lysine residues of histones, thereby silence all heterochromatin-like regions including telomeres, rDNA, and the hidden mating type loci HML/HMR (Hidden MAT Left/ (Hidden MAT Right). ^143^ In the *S. cerevisiae* NAD+ salvage pathway, nicotinamide is recycled from NAD+ by the Sir2 mediated deacetylation reaction.

The nicotinamidase (Pnc1) catalyzes the conversion of nicotinamide to nicotinic acid, which is further converted to nicotinamide mononucleotide by the enzyme nicotinamide phosphoribosyltransferase (Npt1). Isonicotinamide (a compound similar in shape and electrical properties to nicotinamide) was shown to elevate intracellular levels of NAD+ through the yeast NAD+ salvage pathway, leading to an increase in Sir2 activation. This, in turn, enahnce normal silencing at the rDNA locus in yeast and extension of yeast RLS. ^144^

### Parishin

Parishin is a phenolic glucoside isolated from *Gastrodia elata*, a Chinese traditional medicinal plant. Treatment with parishin significantly increased cell viability under oxidative stress and extended the replicative life span of K6001 yeast. Parishin treatment significantly induced the expression of levels of SIR2 and SOD activity, while preventing the accumulation of ROS and lipid peroxidation. However, Parishin did not increase the RLS of *sod1Δ*, *sod2Δ*, *uth1Δ*, and *skn7Δ* mutants of K6001 yeast.

Treatment with parishin suppressed the expression of several TOR signaling pathway-related genes such as TORC1, ribosomal protein S26A (RPS26A), and ribosomal protein L9A (RPL9A). In addition, parishin significantly diminished the gene expression levels of *RPS26A* and *RPL9A* in *uth1Δ* mutant, as well as in *uth1 Δsir2Δ* double mutants. Also, parishin remarkably suppressed the TORC1 gene expression in uth1 mutants. These study results indicate that parishin exerts its anti-aging activities by modulating Sir2/Uth1/TOR signaling pathway. ^131^

### Phloridzin

Xiang, L. *et al.*, investigated the anti-aging effects of apple polyphenol, phloridzin, using yeast RLS model. Phloridzin treatment significantly increased the percentage viability of yeast cells exposed to hydrogen peroxide. It was also demonstrated that phloridzin treatment led to increased expression of SOD1/2 and SIRT1 genes as well as the activity of superoxide dismutase enzyme. Altogether, it was suggested that the anti-aging effects of phloridzin are mediated through the regulation of expression of SIRT1 in budding yeast. ^129^

### Rapamycin

Eukaryotic cells growth and proliferation is regulated by an evolutionarily conserved kinase, the target of rapamycin (TOR). It has been shown that the inactivation of TOR signaling pathway leads to lifespan extension in different eukaryotic model organisms. The eukaryotic nucleolus is rich in ribosomal DNA (rDNA) that is composed of multiple tandem repeats of rRNA genes (100-200 copies rDNA repeats) and the components for ribosome assembly. Inside the nucleolus, the transcription of rDNA produces precursor rRNA that in turn will be processed further and become associated with ribosomal proteins to form preribosomal subunits.

Sir2 is a histone deacetylase enzyme involved in silencing the transcription at the rDNA locus to enhance yeast life span. ^145^^,^^146^ Nearly 50% of the rDNA repeats are maintained in a silent state partly by the Sir2 protein. ^147^ Using *S. cerevisiae* yeast as model, it was found that rapamycin treatment cause inhibition of the TORC1 complex, resulting in the increased association of Sir2 with ribosomal DNA (rDNA) in the nucleolus. This association of SIR2 with rDNA reduces homologous recombination between rDNA repeats that causes formation of toxic extrachromosomal rDNA circles. Thus, rapamycin treatment-induced TORC1 inhibition signals the stabilization of rDNA locus by promoting the association of Sir2 with rDNA, thereby extending the RLS in *S. cerevisiae.* ^148^

### Resveratrol

Mitochondrial dynamics, the balance between mitochondrial fission and fusion, is critical for cell growth and functioning. Replicative senescence in yeast is characterized by the presence of fragmented mitochondria due to mitochondrial fission rather than fusion, indicating altered mitochondrial dynamics. Wang *et al.* reported that treatment with resveratrol led to a significant reduction in the number of senescent yeast cells with fragmented mitochondria. This indicates that resveratrol’s anti-aging effects possibly occur via modulating the expression of genes associated with mitochondrial dynamics during RLS. ^130^

### Psoralea corylifolia

*P. corylifolia* is a medicinal plant widely used in the traditional medicine systems in India and China. Wang *et al.* showed that the ethanol extract of *P. corylifolia* significantly extended the RLS of yeast. In particular, the n-hexane fraction of the ethanol extract of *P. corylifolia* increased the yeast lifespan by 20%. Interestingly, the n-hexane extract of *P. corylifolia* extended the mean lifespan of the *sir2Δfob1Δ* double mutant strain, indicating that the n-hexane fraction prolongs the RLS in a Sir2-independent pathway. Treatment with *P. corylifolia* did not extend the RLS of the *tor1Δ* mutant, suggesting that Tor1 plays a major role in the extension of RLS by the n-hexane-soluble fraction *P. corylifolia.*

Furthermore, it was reported that, Corylin and neobavaisoflavone are the active compounds in *P. corylifolia* that significantly increased the viability and extended the RLS of yeast. In particular, corylin was found to be more effective in enhancing the yeast RLS compared to neobavaisoflavone. It was also shown that corylin treatment promoted RLS of the *sir2Δfob1Δ* mutant strain but failed to prolong the RLS of the *tor1Δ* mutant, suggesting that corylin exerts life span-extending effects in a Tor1-dependent pathway. Fascinatingly, under CR conditions, corylin could not extend the RLS. In addition, corylin treatment did not significantly influence the CLS of yeast. Gtr1 in *S. cerevisiae* encodes a highly conserved GTPase that is necessary for TORC1 activation and amino acid sensing. The G protein complex Gtr1/Gtr2 activates Tor1 in *S. cerevisiae* in response to signals from amino acids. ^149^ Furthermore, it was also suggested from docking studies that corylin prolongs the yeast RLS by blocking Gtr1 activation. ^132^

Previous studies by Lee MB et al also demonstrated that green tea extract and berberine could strongly shorten the yeast life span, whereas *Pterocarpus marsupium* extract and other mixtures containing *P. marsupium* significantly extended the yeast life span. ^133^ In another study, two sesquiterpene glucosides isolated from the Shenzhou honey peach fruit were also able to extend the RLS of K6001 yeast. Treatment with sesquiterpene glucosides increased the survival rate of yeast under oxidative stress. Besides, treatment with sesquiterpene glucosides could not affect the RLSs of SOD mutant yeast strains with a K6001 background, indicating that the anti-oxidative stress response performs important roles in anti-aging effects of sesquiterpene glucosides. ^123^

### Natural products/plant extracts vs. both CLS & RLS

Some natural products have also been demonstrated to extend both RLS and CLS in yeast model. These natural products include curcumin (a diarylheptanoid), ^150^ Cucurbitacin B (a triterpenoid), ^136^ Gentirigeoside B (a dammaren-type triterpenoid glycoside), ^151^ Gentiopicroside (a secoiridoid glycoside), ^152^ and inkosterone (a phytoecdysteroid). ^153^ Table 3 lists the natural compounds, their concentrations, phytochemical class, type of growth medium used for aging experiments, and their anti-aging mechanisms in different *S. cerevisiae* strain backgrounds.

**Table 3.** List of Natural products extending both CLS & RLS.

Name	Chemical Class	Dose	Yeast Strain	Yeast Growth Medium	Anti-Aging Mechanism	Ref.
Curcumin	Diarylheptanoid	200 & 300 μM	BY4741	SD	• Increased oxidative stress and resulting hormetic effects	^150^
Cucurbitacin B	Triterpenoid	0.1, 0.3, 1 μM	K6001	YPGal	• ↑ ATG32 mRNA levels • ↓ ROS and MDA • ↑SOD activity • ↑ SOD12 and SOD2 mRNA levels • through the regulation of expression of *UTH1* as well as *SKN7.*	^136^
Gentirigeoside B	Dammaren-type triterpenoid glycoside	1, 3, and 10 μM	K6001	SD	• ↑ Enzymatic activities of SOD, CAT, Gpx • ↓ ROS and MDA • ↓ SCH9 mRNA and ↑ mRNA levels of RIM15 and Msn2	^151^
Gentiopicroside	secoiridoid glycoside	1, 3, and 10 μM	K6001 and YOM36	SCM+ 2%glucose+ 2% peptone+1% yeast extract and SD	• ↑ ATG32 mRNA levels • ↑ Activity of SOD, CAT, Gpx • ↓ ROS and MDA	^152^
Inokosterone	Phytoecdysone	0.1, 0.3, 1, 3, and 10 μM	K6001 and YOM36	YPGal and SD	• ↑ Sod • ↓ ROS and MDA levels • ↑ autophagy (especially mitophagy)	^153^

YPD (1% yeast extract, 2% peptone, 2% dextrose); YPGal (1% yeast extract, 2% peptone, 2% Galactose); YPGly (1% yeast extract, 2% peptone, 3% Galactose); SCM (synthetic complete medium). SD (Synthetic defined) medium. Symbols ‘↑’ and ‘↓’ indicate up-regulation/increase and down-regulation/decrease, respectively.

### Curcumin

Curcumin is a biologically active yellow-colored carotenoid compound with potent anti-oxidant and anti-aging properties. Previous studies investigated the replicative and chronological life span-extending effects of curcumin using yeast. It was demonstrated that curcumin significantly increased oxidative stress and enhanced both replicative and chronological life spans of yeast mutants that lacked antioxidant genes ( *SOD1* and *SOD2*) and DNA damage repair gene *RAD52.* Overall, it can be suggested that curcumin exerts anti-aging hormetic effects in yeast. ^150^

### Gentirigeoside B and gentiopicroside

Gentirigeoside B is a triterpenoid glycoside isolated from the Chinese traditional medicinal plant *Gentiana rigescens* Franch. Xiang, L., *et al.* reported that gentirigeoside B significantly extended both the replicative and chronological lifespans of yeast. It was also demonstrated that treatment with gentirigeoside B augmented the activity of antioxidant enzymes (including superoxide dismutase, catalase, and glutathione peroxidase) and reduced oxidative stress markers (ROS and MDA), thereby preventing oxidative stress-induced reduction in the viability of yeast cells. Gentirigeoside-B treated cells also showed downregulation of Sch9 and activation of Rim15 and Msn2 proteins. This indicates that the anti-aging potential of gentirigeoside B is due to its ability to inhibit the TORC1/Sch9/Rim15/Msn signaling. However, gentirigeoside B could not enhance the life span of yeast mutant lacking antioxidant genes ( *sod1∆*, *sod2∆*, *cat1∆*, and *gpx∆*) and age-related genes ( *skn7∆* and *uth1∆*). Therefore, Xiang, L., *et al.* suggested that though treatment with gentirigeoside B might have longevity-promoting effects in humans, the effect may not be effective in those with mutations in endogenous antioxidant enzyme genes. ^151^

In another study, gentiopicroside, a secoiridoid glycoside that was also isolated from *G. rigescens* Franch was reported to extend both the RLS and the CLS of yeast. Gentiopicroside was shown to induce ATG32 gene expression, but could not prolong the RLS and CLS of yeast mutants deficient in ATG32 gene. It was also reported that gentiopicroside enhanced the yeast survival rate under oxidative stress condition by augmenting the activities of enzymatic antioxidants and diminishing the accumulation of ROS and lipid peroxidation. However, gentiopicroside could not affect the RLSs of *sod1Δ*, *sod2Δ*, *uth1Δ*, and *skn7Δ.* Overall, it can be suggested that gentiopicroside’s anti-aging effects are attributed to its ability to ameliorate autophagy and antioxidative stress response. ^152^

### Inokosterone

A recent study, ^58^ isolated a compound, inokosterone from *G. rigescens* Franch and demonstrated that inokosterone can extend both the chronological and replicative life spans. The inokosterone was shown to enhance the survival rate of yeast cells by ameliorating the levels of antioxidant enzymes (e.g., SOD) and preventing accumulation of oxidative stress markers (e.g., ROS and MDA levels). Further, inkosterone could alleviate the autophagy (especially mitophagy) in yeast cells. Likewise, the same study also demonstrated that treatment with inkosterone decreased oxidative stress and enhanced autophagy in mammalian cell lines. Therefore, it can be suggested that inkosterone’s anti-aging effects are mediated through the activation of antioxidant stress response and mitophagy. ^153^

### Emerging trends and future directions

An exponential interest in the pursuit for youthful, vital, and quality of human life have become the key driving forces in the development of novel scientific and technological advancements in the anti-aging field. Recent key developments in anti-aging research include nanocapsulation, nutrigenomics, stem cell therapy, *senescent therapy*, as well as the application of artificial intelligence (AI). These innovations are useful for forecasting the potential of different anti-aging treatments and to develop personalized anti-aging strategies. *Nanocapsulation* of anti-aging compounds ensure their sustainable release at target site improving the therapeutic effectiveness. The development of personalized anti-aging nanoformulations might enhance treatment efficacy with minimal side effects. ^154^^,^^155^ *Nutrigenomics* is an emerging filed that explores how nutrients and dietary factors influence cellular processes. This research focuses on how these factors can modulate age-related gene expression, reduce the risk of age-related diseases, and improve overall quality of life. Understanding gene-diet interactions may allow for the development of precise anti-aging nutritional strategies to promote health and longevity. ^156^ *Stem cell therapy, p*articularly, the use of mesenchymal stem cells that can differentiate into skin cells, make them potentially restore skin elasticity and combat skin-aging. ^157^ *Senotherapy:* There has been a growing interest in the screening, identification, evaluation, and development of potent senotherapeutics including senolytics that selectively kill senescent cells and senostatics/senomorphics that reduce the senescent associated pro-inflammatory phenotype. thereby improving healthspan and potentially reducing the prevalence of chronic conditions. ^158^ *Artificial Intelligence (AI):* The revolutionary development of AI algorithms could help in predicting different age-related biomarkers and the progression of age-linked diseases, such as cancer, cardiovascular diseases, neurodegenerative diseases, diabetes, etc. ^159^ AI's ability to predict diseases related biomarkers offers hope for developing targeted anti-aging therapies and managing the disease risk associated with an aging population.

## Conclusions

Aging is a significant risk factor for the emergence of several chronic human diseases. In order to reduce the pathophysiology of diseases related to aging and to increase the active lifetime of people, aging research is primarily concerned with the identification of new anti-aging therapies. Budding yeast *S. cerevisiae* has been used as a valuable model to evaluate the anti-aging properties of phytochemicals as well as synthetic compounds. Several studies demonstrated that plant extracts/phytochemicals can increase longevity via regulation of various pathways including the TORC1/Sch9/Rim15/Msn signaling pathway, RAS-AC-PKA pathway, Sir2-dependent pathway, NAD+-salvage pathway, Uth1/TOR signaling, and antioxidant stress response pathway as well as autophagy. Notably, some natural products exert their anti-aging potential by enhancing the hormetic response in aging yeast cells.

While many of these studies reported enhancing the yeast cell’s oxidative stress resistance, a few studies have also documented the pro-oxidant effects of various antioxidants, suggesting dose-dependent effects on life span. For example, in the K6001 yeast strain of *S. cerevisiae*, treatment with alpha-tocopherol and coenzyme Q10 enhanced oxidative stress and shortened RLS, showing the potential pro-oxidant effects of antioxidants that may be dose-dependent. ^160^ Therefore, to maximize the therapeutic benefits of antioxidants, it is necessary to evaluate their dose-dependent effects as well as potential pro-oxidant actions. Mounting evidence from anti-aging research using yeast models is invaluable in exploring the CR mimetic effects of natural products like artesunate. ^87^ The discovery of novel plant-derived chemicals with anti-aging activities opens up new opportunities for developing leading medications for the treatment of age-related disorders.

Several factors influence yeast survival during chronological aging, such as the concentration of the primary carbon source (like glucose), different types of other carbon sources (e.g., ethanol and acetic acid), the composition of amino acids in the growth medium, and the pH of the aging culture medium can affect the survival of yeast cell during chronological aging. More than 1000 genes are linked to variations in CLS in *S. cerevisiae,* highlighting significant role of genetic material in understanding the fundamental molecular and cellular processes controlling life span in eukaryotes. Most of the anti-aging research studies reviewed here focus on gene deletion mutant strains, which are associated with some drawbacks. ^161^ For instance, gene deletion mutant strains also harbor a secondary mutation (e.g., auxotrophies lacking additional genes like URA or TRP), resulting in heterogenous populations in over 50 % of the strains. ^162^ This heterogeneity can lead to the misinterpretations of genotype and phenotype associations. Furthermore, all gene deletion mutant strains are typically derived from few laboratory strains, primarily the BY strain. This By strain exhibits extreme phenotypic traits that may not accurately represent the natural diversity of the *S. cerevisiae* species. ^163^

In conclusion, aging research using budding yeast as a model organism has yielded valuable insights into the anti-aging properties of various compounds, and the identification of specific pathways and mechanisms involved contributes to the development of potential anti-aging therapies for improving health span and longevity in humans. Continued research into plant extracts and natural compounds may lead to the identification of promising candidates for fighting age-related disorders in the future.

## Data Availability

No data are associated with this article.
