# Supplementary material for: Exploring the anti-aging potential of natural products and plant extracts in budding yeast Saccharomyces cerevisiae: A review
Source: F1000Res. 2024 Dec 17;12:1265. Originally published 2023 Oct 4. [Version 2] doi: 10.12688/f1000research.141669.2 (PMC11736113; doi:10.12688/f1000research.141669.2)
Supplement: Peer review history

# Reviewer report: Reviewer response for version 2

**DOI:** 10.5256/f1000research.175742.r350456

**Published:** 2025-01-24

**Authors:** Weiwei Dang

**Affiliations:** 1 Huffington Center On Aging and Department of Molecular and Human Genetics, Baylor College of Medicine, Houston, TX, USA

The manuscript titled **"Exploring the Anti-Aging Potential of Natural Products and Plant Extracts in Budding Yeast"** provides an in-depth review of the role of *Saccharomyces cerevisiae* as a model organism in aging research. The key focus areas include:

- **Natural Products and Their Mechanisms:** The review discusses several natural compounds and plant extracts that influence aging pathways in yeast, organized according to their impact on chronological lifespan (CLS) and replicative lifespan (RLS).
- **Molecular Pathways:** Key pathways involved in yeast aging, such as the TOR/Sch9 and RAS/AC/PKA pathways, are extensively reviewed in relation to their conserved roles in both yeast and human aging processes.
- **Experimental Considerations:** The manuscript highlights the strengths and limitations of using yeast as a model, such as its genetic tractability versus its limitations in modeling complex multicellular aging processes​.
- **Emerging Trends:** A new section in the revised manuscript explores novel approaches in anti-aging research, including nanotechnology, artificial intelligence, and nutrigenomics​.
- **Future Directions:** It identifies areas needing further exploration, such as understanding the genetic interactions with natural products and optimizing experimental conditions for better translational outcomes​.

**Strengths of the Manuscript**

1. **Comprehensive Literature Review:** The manuscript effectively compiles and organizes a wide range of studies related to natural products' effects on yeast aging.
2. **Revised Structural Organization:** Following peer reviews, the manuscript now presents data in a more logical flow by categorizing compounds under CLS, RLS, or both​.
3. **Enhanced Figures and Tables:** The revised version includes expanded tables summarizing natural product actions, their sources, dosages, and effects, making the content more accessible​.
4. **Addressing Reviewer Comments:** The authors have been responsive to reviewer feedback, incorporating their suggestions to improve readability and focus​.

**Weaknesses of the Manuscript**

1. **Terminology Issue:** The manuscript refers to CLS and RLS as "patterns" of aging; however, it would be more accurate to refer to them as distinct **models** of yeast aging, reflecting their independent mechanistic underpinnings.
2. **Overlapping Concepts:** While the authors have reorganized content, there are still sections where CLS and RLS mechanisms are described with some redundancy, which could be streamlined further.
3. **Lack of Human Translational Insights:** Although yeast is an excellent model, further discussion on how the findings translate to human aging interventions would improve the manuscript's impact.
4. **Experimental Variability:** The manuscript should emphasize more on the influence of yeast strain background, auxotrophic mutations, and media composition on the reproducibility of findings​.

I hope that the authors can address these remaining weaknesses in a minor revision.

Is the review written in accessible language?

Yes

Are all factual statements correct and adequately supported by citations?

Yes

Are the conclusions drawn appropriate in the context of the current research literature?

Yes

Is the topic of the review discussed comprehensively in the context of the current literature?

Yes

Reviewer Expertise:

Yeast aging, epigenetics, chromatin, stem cell aging, senescence, sirtuins

I confirm that I have read this submission and believe that I have an appropriate level of expertise to confirm that it is of an acceptable scientific standard, however I have significant reservations, as outlined above.

# Reviewer report: Reviewer response for version 2

**DOI:** 10.5256/f1000research.175742.r352761

**Published:** 2025-01-16

**Authors:** Hemagirri Manisekaran

**Affiliations:** 1 Institute for Research in Molecular Medicine (INFORMM), Universiti Sains Malaysia, Pulau Pinang, Malaysia

Summary of the Article

 The paper titled "Exploring the anti-aging potential of natural products and plant extracts in budding yeast *Saccharomyces cerevisiae*: A review" discusses the role of natural products, particularly plant extracts, in combating aging, using the model organism *Saccharomyces cerevisiae*. The authors explore various biochemical pathways related to aging, such as the target of rapamycin (TOR) and protein kinase A (PKA) signaling pathways. The review highlights historical uses of plant-derived substances for medicinal purposes and emphasizes the need for further research into natural products as potential anti-aging therapies.

 Overall Assessment: Yes, the paper is well-structured and presents a comprehensive review of the anti-aging potential of natural products and plant extracts in *Saccharomyces cerevisiae.*

 Comprehensiveness of the Topic: Yes

 The review discusses the topic comprehensively within the context of current literature. It effectively covers various aspects of aging and natural products.

 Factual Statements and Citations: Yes

 All factual statements are correct and adequately supported by citations from relevant literature. The authors have done a commendable job in referencing key studies.

 Accessibility of Language: Yes

 The review is written in accessible language, making it understandable for a wide audience, including those who may not be specialists in the field.

 Appropriateness of Conclusions: Partly

 The conclusions drawn about the potential of natural products as anti-aging therapies are valid but could be more comprehensive. The authors should discuss limitations in current research and propose specific future research directions to enhance their conclusions.

Is the review written in accessible language?

Yes

Are all factual statements correct and adequately supported by citations?

Yes

Are the conclusions drawn appropriate in the context of the current research literature?

Partly

Is the topic of the review discussed comprehensively in the context of the current literature?

Yes

Reviewer Expertise:

Aging

I confirm that I have read this submission and believe that I have an appropriate level of expertise to confirm that it is of an acceptable scientific standard.

# Reviewer report: Reviewer response for version 2

**DOI:** 10.5256/f1000research.175742.r357244

**Published:** 2025-01-15

**Authors:** Marco Eigenfeld

**Affiliations:** 1 Medical University Graz, Graz, Australia

**Abstract**

 The sentence, “The budding yeast *Saccharomyces cerevisiae* has been, and continues to be, an indispensable model organism in the field of biomedical research for discovering the molecular basis of aging *S. cerevisiae* has preserved nutritional signaling pathways (such as the target of rapamycin (TOR)-Sch9 and the Ras-AC-PKA (cAMP-dependent protein kinase) pathways, and shows two distinct aging paradigms chronological life span (CLS) and replicative life span (RLS)”, is overly long, grammatically incorrect, and contains repetitive wording such as "pathways." Breaking this sentence into shorter, more focused sentences would improve readability. For instance, you could restructure it as follows: “The budding yeast *Saccharomyces cerevisiae *has long been an indispensable model organism for studying the molecular basis of aging in biomedical research. It has preserved nutritional signaling pathways, such as the target of rapamycin (TOR)-Sch9 and the Ras-AC-PKA (cAMP-dependent protein kinase) pathways. S. cerevisiae also demonstrates two distinct aging paradigms: chronological life span (CLS) and replicative life span (RLS).”

 Additionally, the abstract would benefit from including specific examples of natural compounds rather than relying on the general term "natural compounds." Incorporating examples like resveratrol, quercetin, or berberine (if relevant) would make the abstract more engaging and provide readers with a clearer understanding of the focus of the study.

**Introduction**

 Rather than simply stating that aging is a major risk factor, I recommend emphasizing the bidirectional relationship: aging promotes diseases, and diseases, in turn, can exacerbate aging. This perspective would provide a more comprehensive understanding of the interplay between aging and disease.

 The sentence starting with “However, life style associated anti-aging …” would benefit from being simplified by dividing it into two sentences. This would enhance readability and make the point more accessible to readers.

 For the statement, “Historically, medicinal plants have been used …”, and the one referencing “The oldest records dating back to around 2600 BCE, …”, please include at least one supporting reference to strengthen the claims and provide credibility to these historical assertions.

 In my opinion, the section beginning with “It is no surprise that traditional …” should be shortened. As it stands, this part is very broad, covering historical, pharmacological, and regulatory topics without a clear narrative structure. Since this section is in the introduction, it should aim to guide the reader toward the paper's central topic. Currently, it lacks a clear connection to anti-aging research, which is the main focus of the paper. Condensing this section and tying it more directly to the context of anti-aging potential in natural products and plant extracts would improve its relevance.

 On the other hand, the final section of the introduction fits perfectly within the context and scope of the paper and provides a clear lead-in to the study. Emphasizing this alignment throughout the introduction would enhance the overall cohesion of the manuscript.

**The budding yeast *Saccharomyces cerevisiae*– A simple eukaryotic model to study aging**

 I would recommend using the term “membrane-enclosed” rather than “membrane-bound” when describing organelles. The term “membrane-bound” could imply that the organelles are physically anchored or fixed in place, which may lead to misinterpretation. “Membrane-enclosed” more accurately conveys that these organelles are surrounded by a membrane without suggesting spatial restriction.

 The size of yeast needs to be more precise. For ovoid cells, it would be important to provide both diameters (length and width) to ensure clarity and scientific accuracy.

 Additionally, the statement “The budding yeast was the first eukaryotic …” and “Most interestingly, yeast share several homologous” requires a proper reference to support this claim. Furthermore “The budding yeast has long been used as a …” as well as in multiple other passages, the authors make claims and refer to other general statements without supporting references. Please revise accordingly.

 To improve readability and avoid redundancy, I suggest varying the starting words in the yeast paragraph. Currently, the repeated use of “The” and “Yeast” at the beginning of multiple sentences makes the section feel monotonous. In the first paragraph: The … Yeast … Yeast … The … The … The … The … The … The … Interestingly … The; Consider using alternative phrasing or reworking sentence structures to create a more engaging flow.

 The section starting with “These features make yeast an ideal model organism …” and the subsequent discussion of its limitations needs to be restructured. As it currently stands, the focus shifts heavily toward the limitations, leaving a somewhat negative impression about the use of yeast as a model organism. I recommend balancing the argument by first emphasizing the unique advantages of yeast and then framing the limitations more constructively, such as highlighting areas where complementary models or additional approaches may be necessary, or why yeast is ideal for anti-aging substance tests. This would ensure the argument remains balanced and aligns with the overall positive perspective on yeast as a valuable tool in aging research.

**Replicative lifespan (RLS) and chronological lifespan (CLS)**

 The section discussing replicative lifespan (RLS) and chronological lifespan (CLS) could benefit from some adjustments to improve clarity and relevance. In the second line, the authors mention the number of bud scars without explaining that the bud scar remains after cytokinesis. Including this detail would help readers who might not already be familiar with this concept.

 In the sentence beginning “In old mother cells, the actin cytoskeleton...,” it would be helpful to include a reference for this statement, as well as for the subsequent sentence. These references are particularly important because they provide context for understanding the limited RLS of yeast, which is otherwise considered theoretically immortal.

 The following two paragraphs delve deeply into extrachromosomal rDNA circles (ERCs). It’s worth considering whether this level of detail is necessary. How do these paragraphs connect to the paper’s main topic and title? If ERCs are relevant to the anti-aging potential of the extracts discussed later, this connection should be made explicit. Otherwise, these paragraphs might be overly detailed and disrupt the flow of the paper. If they are not crucial, I would suggest removing them to streamline the narrative.

 The sentence “In worms, flies, and mice, the key...” and similar statements, such as “In addition, stationary phase cells show...,” lack references. Since this is a review article, it’s essential to provide references for such claims. While this issue recurs throughout the text, I won’t list every instance here. I strongly encourage the authors to carefully review the entire manuscript and ensure all statements are properly supported by references. This is especially important in a review, where all information should be accurately cited rather than assumed to be common knowledge.

 The details on diauxic effects and growth curves may not be essential for explaining CLS. Overall, the CLS section, like the RLS section, feels quite long and lacks a clear connection to the review’s main topic. I recommend condensing these sections and focusing more on how they relate to the central theme. There are already numerous review papers on yeast lifespan that could be referenced instead of including extensive background information here. This would help maintain a logical flow and keep the focus on the specific objectives of the paper.

**Conserved nutrient sensing aging regulatory pathways in budding yeast**

 This section is well-written and provides a concise summary of key knowledge that is essential for understanding the mechanisms discussed later in the manuscript.

**Anti-aging studies of natural products and plant extracts in the budding yeast *S. cerevisiae* as a model**

 On page 10 of the review, the main topic is finally introduced, addressing a fascinating and novel area of knowledge. However, there are some aspects that could benefit from improved clarity and scientific rigor. For instance, the opening sentence refers to “several,” but no examples or references are provided. To ensure scientific accuracy and strengthen the credibility of the text, including specific examples or citations would be helpful.

 Similarly, the sentence beginning with “Natural compounds, which…” would benefit from the inclusion of examples, as it feels incomplete. In the subsequent sentence, the discussion becomes more specific by focusing on plant-isolated compounds, which creates some inconsistency. If the terms “natural compounds” and “plant-isolated compounds” are intended to be synonymous, it would be better to use just one term consistently throughout this section to avoid confusion.

 Later, the word “various” is used, which lacks precision. Since eight mechanisms are mentioned, it would be clearer to state “eight mechanisms” explicitly. Such specificity would enhance the scientific rigor of the text and make it easier for readers to follow.

**Natural products/plant extracts vs. CLS**

 The authors begin again with the term “several studies” but fail to reference specific studies to support the statement that these studies explored the mentioned topic. Providing appropriate citations would greatly strengthen the credibility and scientific rigor of the manuscript.

 Furthermore, in Table 1, the authors appear to assume that all yeasts are *Saccharomyces *yeasts, which is not accurate. It would be important to specify that the yeasts in question are *Saccharomyces* strains or provide more precise terminology to avoid potential misunderstandings.

 Additionally, throughout the manuscript, there is a lack of clarity when referring to yeast. The authors are encouraged to use more specific terminology, such as *Saccharomyces cerevisiae*, whenever applicable. This will not only improve precision but also help readers better understand the scope and focus of the discussion.

 In following I will not include all subsection names:

 In the section on “Acetyl L-carnitine,” the authors refer to “Researchers have shown…” without citing specific studies or providing detailed evidence. Including references to support this statement would add credibility and depth to the discussion. Additionally, there is a typographical error in this section where “Ca2+” is written without the superscript. Correcting this will improve the professionalism and accuracy of the presentation.

 In the astaxanthin section, the authors refer to a study by Sudarshan et al., but the reference number is missing at the end of the sentence. Including the reference number is important for accessibility, as it makes it easier for readers to locate the source without having to cross-reference the name with the reference list. The same issue arises at the end of the section, where a reference number should also be included.

 Regarding the compound artesunate, its inclusion raises an important question: if the compound is semi-synthetic, why is it included in a paper focusing on compounds extracted from natural sources? This point needs clarification to align the discussion with the scope of the paper. Additionally, this section suffers from a lack of references. While the text mentions “Previous studies have shown” in the plural form, only one reference is provided at the end. To maintain scientific rigor, it is necessary to either cite multiple studies or adjust the wording to reflect the actual number of cited works.

 In the section discussing Betulinic acid, the authors state that it is present in some plant species but do not provide examples. Naming specific plant species where Betulinic acid is found would add valuable detail and context for the readers, enhancing the depth of the discussion.

 In the section focusing on citrus, the issue of plural wording remains unresolved. If writing in plural, more than one reference must be provided to substantiate the claim. Additionally, it would be helpful to explain why ROS accumulation is prevented, as this adds clarity and context to the discussion.

 In the section on “4-N-furfurylcytosine,” the authors should clarify why viability is increased and why mitochondrial activity is boosted. These are important mechanistic details that should not be omitted. Furthermore, the source of isolation for this compound is not mentioned, which detracts from the manuscript’s alignment with the scope of natural products.

 The section on “Galactan exopolysaccharide” refers to a study without citing it. Proper citation is necessary here, and the mechanism of action for this compound should also be explained to provide a deeper understanding of its effects.

 In the discussion of Ginsenoside, the authors use the plural form to refer to studies but cite only a single reference. This inconsistency should be corrected either by providing additional references or by adjusting the language to the singular form.

 The section on “Lithocholic acid” has a similar reference issue, as the authors fail to include the reference number for the study by Beach et al.

 In the section on Magnolol, it is unclear whether the reference cited is the only available one. Including a second reference, if available, would enhance the accuracy and credibility of this section. More broadly, sections that rely on a single reference would benefit from being expanded with additional supporting studies.

 For the sections on Myricetin and Spermidine, the citation issue persists as above, with references not being properly cited. The same applies to Quercetin, and additionally, the discussion on why ROS levels are reduced is missing and should be included to strengthen the argument.

 The section on Almond extract also has issues with plural language and missing references. Moreover, it is unclear why the alphabetical order begins again in this section, which disrupts the structure and flow of the manuscript.

 The inclusion of a section titled “Natural products/plant extracts vs. RLS” in the list is confusing and seems out of place. Is maybe this a new header? Then please increase text size. The same with RLS&CLS.

 The alphabetical order restarts here without explanation, creating the impression of rushed revisions. The manuscript would benefit from a thorough restructuring to ensure consistent organization. The authors should revise the manuscript as a whole to address these recurring issues with references, singular/plural discrepancies, and the alphabetical ordering of sections.

**Overall comments**

 To improve the clarity and coherence of the manuscript, I recommend restructuring the listing of compounds in a systematic and consistent way. Each compound should be presented with the following elements:

1. **Source**: Clearly state from which natural source the compound is isolated. This ensures alignment with the scope of the manuscript and provides essential context for each compound.
2. **Effect**: Describe the specific biological or physiological effect of the compound.
3. **Mechanism**: Provide an explanation of how the observed effect is achieved, ideally supported by evidence from studies.

Currently, many compounds are missing one or more of these critical aspects, which makes the listing incomplete and inconsistent. Ensuring all three elements are addressed for each compound will significantly enhance the comparability and scientific rigor of the discussion.

 Moreover, some compounds have been discussed in the context of key pathways such as TORC1/Sch9/Rim15/Msn signaling, RAS-AC-PKA pathway, Sir2-dependent pathway, NAD+-salvage pathway, and Uth1/TOR signaling. For example Acetyl-L-carnitine, astaxanthin, betulinic acid, galactan exopolysaccharide, magnolol, myricetine. These pathways appear central to the manuscript’s main point, yet their discussion is sparse and insufficiently integrated. To better support the main argument, each compound should be explicitly linked to one or more of these pathways whenever applicable. This will tie the individual sections together and link to the introduction of the pathways.

 After reviewing the manuscript again, the section on ERCs is unclear, as it is never referenced in the later context. Additionally, RLS and CLS are described in excessive detail, which seems unnecessary for the later sections. I would like to see more focus on how the compounds impact RLS and CLS. These details, which would provide valuable new insights for a review, are missing from the compound descriptions. Specifically, quantitative data such as how many additional buddings are possible due to the compounds or how many hours the CLS is extended by their antioxidative effects would greatly enhance the manuscript.

Is the review written in accessible language?

Yes

Are all factual statements correct and adequately supported by citations?

Partly

Are the conclusions drawn appropriate in the context of the current research literature?

Yes

Is the topic of the review discussed comprehensively in the context of the current literature?

Partly

Reviewer Expertise:

Investigating the mechanisms of yeast aging and yeast physiology, with afocus on the interplay between aging and protein synthesis.

I confirm that I have read this submission and believe that I have an appropriate level of expertise to confirm that it is of an acceptable scientific standard, however I have significant reservations, as outlined above.

# Reviewer report: Reviewer response for version 1

**DOI:** 10.5256/f1000research.155136.r253397

**Published:** 2024-03-26

**Authors:** John Hartman

**Affiliations:** 1 Genetics, University of Alabama-Birmingham, Birmingham, AL, 35294-0024, USA

The authors may want to consider more precise and directional adjectives, such as enhance, suppress, augment, alleviate, etc. Use of the word ‘ameliorate’ to qualify various biological effects, seemed vague, inconsistent, and sometimes incorrect. My suggestion would be to carefully proof-read again for grammar in places but also for biological clarity, for example, about relationships between TOR signaling and respiration.

 Overall, I thought the review topic was very interesting -  the authors have assembled a collection of papers about natural products (NPs) where biological activities have been studied using paradigms (RLS and CLS) of yeast aging. 

 The abstract and introduction could be greatly enhanced by removing much of the philosophical and physiological descriptions of aging, how it's fundamental to several diseases, and the utility and experimental benefits of model organisms, all of which have been made many times and are thus an unnecessary distraction. If condensed to a couple sentences and citations there would be much space cleared to focus more on the topic at hand which is already very rich and extensive and could stand to be expanded upon and further detailed.

 Fig. 1 is awkward IMO (in a BioRender sort of way) and it's not really needed. To regain that space and maintain reader attention, a simple brief description of RLS and CLS would do (with just a few of many possible citations that could support the rare reader who may not be already familiar). 

 It would be helpful to organize the paper according to which NPs were studied in the context of RLS, CLS, or both. The models are quite different, though they involve related mechanisms as the authors point out. Clarifying and distinguishing (or unifying) between the RLS/CLS models over the course of the MS would be of interest (more to come on this and other factors that could be better organized in order to increase the impact of the work).

 Fig. 2 is helpful, but should be expanded with regard to the genes and pathways (and the page space) and doesn't need to be rendered as an actual cell if removing the picturesque constraints of the cell and organelles would provide more flexibility to communicate important information about the literature.

 The "conserved nutrient sensing aging regulatory pathways" section is critical and could be expanded substantially. "Conserved" presumably means "between" yeast and humans? This section seems woefully under-cited. If possible, all of the genes mentioned in the NP papers should be incorporated into this section and Fig. 2 and related to one another, which would ultimately help to relate the NPs to each other regarding their influences on cellular aging. There is an opportunity here to relate the yeast pathways to the human pathways (i.e. to relate the conserved pathways), and ultimately to suggest how some of the NPs, which influence yeast aging, could be related to benefits they are thought to putatively have in humans. I think that opportunity wasn't really capitalized on but would be great if the authors could. This would provide demonstrations for the significance of the topic and the importance of the review as a starting point to increase knowledge about relationships between NPs, yeast aging models, and human age-related disease.

 The "Anti-Aging studies "of NPs" in S. cerevisiae" section should mention NPs  in the section subtitle (just for clarity). 

 Rather than just a list of NPs - this section would be much more useful if organized by important factors. I'm not sure exactly how to do it, but a table would very helpful for handling this aspect. Strategies that could be used to organize this section include alphabetical order, whether influences are on CLS, RLS, or both; class of compounds; relevant sources of the compounds (foods, alternative medicines, natural metabolism, microbiome, etc); whether they have known or suspected effects on human health; MOA or genes and pathways they are thought to act or interact with; whether the NPs are easily obtained for research purposes (i.e. where to buy them); other factors that the authors may be aware of. 

 A table that synthesizes and distills the important factors relevant to each NP should be created (as also suggested by Reviewer 1). The table should have a field dedicated to each factor (see a few suggestions in paragraph above) and have concise record entries for each NP - one of the fields should be for the references for convenient lookup of information in the table context. If provided as a spreadsheet, it would be particularly useful.

 Just my personal opinion, but I didn't see how descriptions of the advantages of yeast, emerging trends, cutting edge techniques, or future directions would be as helpful as more detail and better organization about the NPs, in-depth summary about the literature where yeast have been used to study them, and if possible where they are are also relevant to human and/or specific age-related diseases. 

 About "conclusions drawn being 'partly' supported" there can be large effects on CLS deriving from differing auxotrophic background of yeasts strain and media recipes. Things like glucose concentration, whether the media is buffered, amino acid compositions, the presence of ammonium sulfate, rich vs. synthetic/defined media, the list goes on. The interactions between the NPs and genetic background was discussed  in the review, but these same types of interactions exist with auxotrophic mutations and media composition (which can vary dramatically between studies). This fact should be briefly mentioned in the manuscript and ideally accounted for in the proposed table by indicating the strain, auxotrophic background and media used in the studies employing particular NPs. Additives like H2O2 that are used to enhance characterization are mentioned in places and could also be accounted for in the table.

 I appreciate the authors' efforts and insights hope they find this feedback useful.

Is the review written in accessible language?

Yes

Are all factual statements correct and adequately supported by citations?

Partly

Are the conclusions drawn appropriate in the context of the current research literature?

Partly

Is the topic of the review discussed comprehensively in the context of the current literature?

Partly

Reviewer Expertise:

yeast genetics, human disease models, CLS

I confirm that I have read this submission and believe that I have an appropriate level of expertise to confirm that it is of an acceptable scientific standard, however I have significant reservations, as outlined above.

## References

[1] : High-resolution yeast quiescence profiling in human-like media reveals complex influences of auxotrophy and nutrient availability. *Geroscience* .2021;43(2) : 10.1007/s11357-020-00265-2 941-964 10.1007/s11357-020-00265-2 33015753 PMC8110628

[2] : Gene-nutrient interaction markedly influences yeast chronological lifespan. *Exp Gerontol* .2016;86: 10.1016/j.exger.2016.04.012 113-123 10.1016/j.exger.2016.04.012 27125759 PMC5079838

[3] : Genetic interaction profiles of regulatory kinases differ between environmental conditions and cellular states. *Mol Syst Biol* .2020;16(5) : 10.15252/msb.20199167 e9167 10.15252/msb.20199167 32449603 PMC7247079

# Response

**Published:** 2024-12-08

**Authors:** Tewin Tencomnao

**Affiliations:** Faculty of Allied Health Sciences, Chulalongkorn University, Thailand

The authors may want to consider more precise and directional adjectives, such as enhance, suppress, augment, alleviate, etc. Use of the word ‘ameliorate’ to qualify various biological effects, seemed vague, inconsistent, and sometimes incorrect. My suggestion would be to carefully proof-read again for grammar in places but also for biological clarity, for example, about relationships between TOR signaling and respiration.

***Response***: We thank you for your suggestions on the precise usage of adjectives and grammar in this manuscript. As suggested, we have carefully proofread the document and considered the usage of more precise and directional adjectives throughout the revised manuscript.

 Overall, I thought the review topic was very interesting - the authors have assembled a collection of papers about natural products (NPs) where biological activities have been studied using paradigms (RLS and CLS) of yeast aging. 

 The abstract and introduction could be greatly enhanced by removing much of the philosophical and physiological descriptions of aging, how it's fundamental to several diseases, and the utility and experimental benefits of model organisms, all of which have been made many times and are thus an unnecessary distraction. If condensed to a couple sentences and citations there would be much space cleared to focus more on the topic at hand which is already very rich and extensive and could stand to be expanded upon and further detailed.

***Response:*** As suggested by the reviewer, the philosophical and physiological descriptions of aging are removed from the abstract and introduction of the revised manuscript. Additionally, the review has been focused on the natural products (please see Introduction).

 Fig. 1 is awkward IMO (in a BioRender sort of way) and it's not really needed. To regain that space and maintain reader attention, a simple brief description of RLS and CLS would do (with just a few of many possible citations that could support the rare reader who may not be already familiar). 

***Response:*** As suggested by the reviewer, we have included additional information on CLS and RLS along with their respective citations. However, we believe that inclusion of Figure 1 may help readers, particularly budding yeast researchers working on anti-aging research, and facilitate an easier understating of the concepts of CLS and RLS.

 It would be helpful to organize the paper according to which NPs were studied in the context of RLS, CLS, or both. The models are quite different, though they involve related mechanisms as the authors point out. Clarifying and distinguishing (or unifying) between the RLS/CLS models over the course of the MS would be of interest (more to come on this and other factors that could be better organized in order to increase the impact of the work).

***Response:*** We appreciate your insightful suggestion and we have reorganized the anti-aging effects of natural products according to the relevance of CLS or RLS or both. We do believe that this reorganization further enhances the impact of work.

 Fig. 2 is helpful, but should be expanded with regard to the genes and pathways (and the page space) and doesn't need to be rendered as an actual cell if removing the picturesque constraints of the cell and organelles would provide more flexibility to communicate important information about the literature.

***Response:*** As suggested by the reviewer, Figure 2 is improved in the revised manuscript.

 The "conserved nutrient sensing aging regulatory pathways" section is critical and could be expanded substantially. "Conserved" presumably means "between" yeast and humans? This section seems woefully under-cited. If possible, all of the genes mentioned in the NP papers should be incorporated into this section and Fig. 2 and related to one another, which would ultimately help to relate the NPs to each other regarding their influences on cellular aging. There is an opportunity here to relate the yeast pathways to the human pathways (i.e. to relate the conserved pathways), and ultimately to suggest how some of the NPs, which influence yeast aging, could be related to benefits they are thought to putatively have in humans. I think that opportunity wasn't really capitalized on but would be great if the authors could. This would provide demonstrations for the significance of the topic and the importance of the review as a starting point to increase knowledge about relationships between NPs, yeast aging models, and human age-related disease.

***Response:*** Thank you for your insightful suggestion. The section entitled “conserved nutrient-sensing aging regulatory pathways” has been revised with sufficient citations for each of the singling pathways discussed in the review.

 The "Anti-Aging studies "of NPs" in S. cerevisiae" section should mention NPs in the section subtitle (just for clarity). 

***Response:*** As suggested, the tile of the section has been changed to “Anti-aging studies of natural products and plant extracts in the budding yeast S. cerevisiae as a model.”

 Rather than just a list of NPs - this section would be much more useful if organized by important factors. I'm not sure exactly how to do it, but a table would very helpful for handling this aspect. Strategies that could be used to organize this section include alphabetical order, whether influences are on CLS, RLS, or both; class of compounds; relevant sources of the compounds (foods, alternative medicines, natural metabolism, microbiome, etc); whether they have known or suspected effects on human health; MOA or genes and pathways they are thought to act or interact with; whether the NPs are easily obtained for research purposes (i.e. where to buy them); other factors that the authors may be aware of. 

***Response:*** We thank you for your valuable suggestions on the organization of natural products. As suggested, the section on the natural products and plant extracts has been reorganized, with the anti-aging studies now arranged alphabetically in the revised version of the manuscript.

 A table that synthesizes and distills the important factors relevant to each NP should be created (as also suggested by Reviewer 1). The table should have a field dedicated to each factor (see a few suggestions in paragraph above) and have concise record entries for each NP - one of the fields should be for the references for convenient lookup of information in the table context. If provided as a spreadsheet, it would be particularly useful.

***Response:*** Thank you for your suggestion. A table summarizing the natural products and plant extracts including their source, chemical class, dosage used, growth medium used for aging studies, yeast strain used, and their anti-aging mechanisms, has been included in the revised manuscript. Please refer to table 1, 2, and 3

 Just my personal opinion, but I didn't see how descriptions of the advantages of yeast, emerging trends, cutting edge techniques, or future directions would be as helpful as more detail and better organization about the NPs, in-depth summary about the literature where yeast have been used to study them, and if possible where they are also relevant to human and/or specific age-related diseases. 

***Response: *** We thank you for your insightful suggestions on the importance of adding in depth literature on the natural products and the reorganization of the natural products section. We have reorganized the revised manuscript and included an in-depth summary of natural products. 

 About "conclusions drawn being 'partly' supported" there can be large effects on CLS deriving from differing auxotrophic background of yeasts strain and media recipes. Things like glucose concentration, whether the media is buffered, amino acid compositions, the presence of ammonium sulfate, rich vs. synthetic/defined media, the list goes on. The interactions between the NPs and genetic background was discussed in the review, but these same types of interactions exist with auxotrophic mutations and media composition (which can vary dramatically between studies). This fact should be briefly mentioned in the manuscript and ideally accounted for in the proposed table by indicating the strain, auxotrophic background and media used in the studies employing particular NPs. Additives like H2O2 that are used to enhance characterization are mentioned in places and could also be accounted for in the table.

**Response: **We briefly mentioned in the manuscript how different factors such as carbon sources, amino acid composition, pH of the growth medium affects the CLS. Additionally, we have included a table that accounts for the strain background, media used in anti-aging studies of natural products/plant extract.

# Reviewer report: Reviewer response for version 1

**DOI:** 10.5256/f1000research.155136.r212350

**Published:** 2023-10-24

**Authors:** Srinivasa Rao Sirasanagandla

**Affiliations:** 1 Department of Human & Clinical Anatomy, College of Medicine & Health Sciences, Sultan Qaboos University, Muscat, Oman

The current review presents a summary of aging and the use of *Saccharomyces cerevisiae* as a model organism for anti-aging research. It's informative and well-structured, making it suitable for readers interested in the topic. Here are a few suggestions for elevating the quality of this review.

- In the abstract, authors should provide a clear overview of the research focus, its significance, and what readers can expect from the review. Begin with a clear statement of the objective or focus of the research. For example, "This review aims to explore the anti-aging properties of several natural products and phytoextracts using the budding yeast *Saccharomyces cerevisiae* as a model organism." Explain the significance of studying aging and anti-aging research. Highlight the Contribution. Include a statement about what readers can expect from the review.
- In the introduction sections, please cite the figures 1 and 2.
- It would be more informative if authors had provided a table summarizing the action of natural products and/or plant extracts.
- The authors should also highlight the advantages and disadvantages of using yeast as a model for anti-aging research.
- The authors should include a section on emerging trends that focuses on identifying emerging trends and cutting-edge techniques in the study of anti-aging compounds.
- A distinct section focusing on future directions that need further exploration in the field should also be added.

Is the review written in accessible language?

Yes

Are all factual statements correct and adequately supported by citations?

Yes

Are the conclusions drawn appropriate in the context of the current research literature?

Yes

Is the topic of the review discussed comprehensively in the context of the current literature?

Yes

Reviewer Expertise:

Natural compounds against the environmental pollutant induced toxicity, radiological anatomy, morphological variations

I confirm that I have read this submission and believe that I have an appropriate level of expertise to confirm that it is of an acceptable scientific standard.

# Response

**Published:** 2024-12-08

**Authors:** Tewin Tencomnao

**Affiliations:** Faculty of Allied Health Sciences, Chulalongkorn University, Thailand

- In the abstract, authors should provide a clear overview of the research focus, its significance, and what readers can expect from the review. Begin with a clear statement of the objective or focus of the research. For example, "This review aims to explore the anti-aging properties of several natural products and phytoextracts using the budding yeast *Saccharomyces cerevisiae* as a model organism." Explain the significance of studying aging and anti-aging research. Highlight the Contribution. Include a statement about what readers can expect from the review.

***Response:*** We thank reviewer for the insightful comment on the abstract. We have revised the abstract by incorporating a clear overview of the research focus and the significance of the aging and anti-aging research.

  

- In the introduction sections, please cite the figures 1 and 2.

***Response:*** We apologies for overlook in not citing the figures. We have cited Figures 1 and 2 at appropriate sections of the revised manuscript.

  

- It would be more informative if authors had provided a table summarizing the action of natural products and/or plant extracts.

***Response:*** As suggested by the reviewer, we have summarized the anti-aging molecular mechanisms of actions of natural products and plant extracts in Tables 1, 2, and 3.

  

- The authors should also highlight the advantages and disadvantages of using yeast as a model for anti-aging research.

***Response:*** **“**The main advantages of using the yeast *Saccharomyces cerevisiae* in aging research are that it is a unicellular eukaryote that exhibits characteristic features such as rapid growth, inexpensive, and ease of genetic manipulation, allowing to conduct experiments quickly and study the effects of specific genes on aging. Several of the cellular processes and metabolic pathways, including aging, are conserved between *S. cerevisiae* and humans. These features make yeast an ideal model organism for the high-throughput screening of identifying genes and chemical compounds associated with aging. However, the use of yeast has some limitations. For example, its unicellular nature limits its ability to model complex multicellular organisms and tissue-specific aging processes in humans. Additionally, some post-translational modifications that occur in humans do not occur in yeast, and yeast metabolism significantly differs from humans, which may limit applicability if certain findings to human aging research.” This information has been included in the section titled “The budding yeast *Saccharomyces cerevisiae* – A simple eukaryotic model to study aging” in the revised version of the manuscript.

  

- The authors should include a section on emerging trends that focuses on identifying emerging trends and cutting-edge techniques in the study of anti-aging compounds.

***Response: *** We thank reviewers for their valuable suggestion on the necessity of adding a section on the emerging trends in the study of anti-aging compounds. As suggested, a separate section is included in the revised manuscript.

- A distinct section focusing on future directions that need further exploration in the field should also be added.

***Response*** *: *A separate section compiling both emerging trends and future developments in the anti-aging research are included in the revised manuscript.
